# Synthetic Integrin-Targeting Dextran-Fc Hybrids Efficiently Inhibit Tumor Proliferation *In Vitro*

**DOI:** 10.3389/fchem.2021.693097

**Published:** 2021-07-22

**Authors:** Hendrik Schneider, Simon Englert, Arturo Macarrón Palacios, Jorge Alberto Lerma Romero, Ataurehman Ali, Olga Avrutina, Harald Kolmar

**Affiliations:** Institute for Organic Chemistry and Biochemistry, Technical University Darmstadt, Darmstadt, Germany

**Keywords:** integrins, RGD peptide, dextran, drug delivery and targeting, multimerization

## Abstract

Herein, we present the design, synthesis, and biological evaluation of novel integrin-targeting molecular hybrids combining RGD peptides and a potent cytotoxin presented on dextran polysaccharides. Based on an aglycosylated Fc as a centerpiece, endosomal-cleavable cytotoxic agent monomethyl auristatin E (MMAE) and dextran as multimerization site were covalently connected by two bioorthogonal enzyme-mediated reactions site-specifically. Decoration of dextran with cyclic RGD peptides, introduced by copper “click” reaction, resulted in the final constructs with the potential to kill integrin-overexpressing tumor cells. We found that these modifications had little impact on the stability of the Fc scaffold and the RGD-bearing construct showed good binding properties of αvβ3-expressing U87MG cells. Furthermore, the construct showed a remarkable antiproliferative activity. These results demonstrate the general capability of our design to provoke receptor-mediated endocytosis upon binding to the cellular surface, followed by endosomal cleavage of the linkage between Fc-dextran and MMAE and its subsequent release. Our approach opens new avenues to transcribe small molecule binders into tailor-made multimeric molecular hybrids with antitumor potential.

## Introduction

Over the last decades, specific targeting of cancer cells has become an increasingly important issue in the development of novel approaches to antitumor therapy. Numerous strategies have been proposed to reach this long-sought goal, one of them being the combination of unspecific cytotoxic drugs with a targeting moiety (Borsari et al., 2020). In particular, antibody–drug conjugates (ADCs) became popular as vehicles relying on immunoglobins, which bind to overexpressed antigens on cancer cells and therefore facilitate the delivery of cytotoxic agents (Khongorzul et al., 2020). Besides these large and complex architectures, targeting modules based on small molecules and peptides have also been developed (Krall et al., 2013).

Within the range of potential targets for anticancer therapy, integrins have gained special interest due to their importance for tumor progression (Nieberler et al., 2017). Being the key actors in cell–cell and extracellular matrix communication, integrins are important regulators of cellular processes such as adhesion, proliferation, apoptosis, and migration (Humphries et al., 2006; Nieberler et al., 2017). These glycosylated transmembrane proteins are heterodimers; a pool of 18 α-subunits and 8 β-subunits results in at least 24 variants in humans (Yousefi et al., 2021). Expression levels of several integrin subtypes, most prominently αvβ3, have been reported to be elevated in solid tumors, for example, melanoma, breast, pancreatic cancer, and glioblastoma, as well as in tumor blood vessels (Desgrosellier and Cheresh, 2010). Since the discovery of the binding motif arginine–glycine–aspartic acid (RGD) in the natural ligand fibronectin (Pierschbacher and Ruoslahti, 1984), much effort has been directed to the development of improved integrin binders based on this triad sequence. The selectivity for tumor-relevant subtypes and activity was enhanced by cyclization, for example, head-to-tail macrocyclization (Aumailley et al., 1991), and incorporation of diketopiperazine (Marchini et al., 2012). Further progress was achieved by the introduction of d-amino acids (Aumailley et al., 1991) and *N*-methylation (Dechantsreiter et al., 1999). These improvements culminated in the development of the integrin antagonist cilengitide *cyclo*[RGDf(NMe)V] (Dechantsreiter et al., 1999) which unfortunately failed in clinical trials (Tucci et al., 2014). Paradoxically, cilengitide as an integrin agonist induced tumor growth and angiogenesis at low nanomolar plasma concentrations which were reached in most clinical trials during the drug administration schedule (Reynolds et al., 2009). This could be attributed to the finding that cyclic RGD antagonists are capable of promoting a major conformational change in the integrin αvβ3 receptor that prompts it to adopt a high-affinity ligand-binding state (Takagi et al., 2002), thus suggesting that a high local concentration of the ligand is required to avoid these unwanted effects.

Alternatively, RGD-containing peptides can be employed as a tumor-homing module in targeted drug delivery to possibly negate the effects of the concentration-dependent ambivalence. The respective strategies have already been realized by either conjugation of RGD-containing integrin binders to cytotoxic drugs like paclitaxel (Colombo et al., 2012), doxorubicin (Burkhart et al., 2004), cryptophycin (Nahrwold et al., 2013), and monomethyl auristatin E (MMAE) (Lopez Rivas et al., 2019) or genetic fusion to proteins such as tumor necrosis factor-α (TNF-α) (Zarovni et al., 2004). Compared to the solitary ligands, these constructs in general displayed similar binding capabilities to the target integrins and, furthermore, improved pharmacological properties of the conjugated payloads. Besides targeted drug delivery, RGD-containing peptides have also shown their potential for tumor imaging, for example, with radioisotopes as cargo for positron emission tomography (PET) (Chen et al., 2016). In order to enhance the affinity of RGD ligands and also the rate of specific internalization (Sancey et al., 2009; Kemker et al., 2020), multimerized RGD constructs were synthesized. Different platforms for the multimeric presentation were used, among them were polymers (Komazawa et al., 1993), peptides (Thumshirn et al., 2003; Sancey et al., 2009), antibodies (Kok et al., 2002), and liposomes (Wu et al., 2020).

**FIGURE 1:**
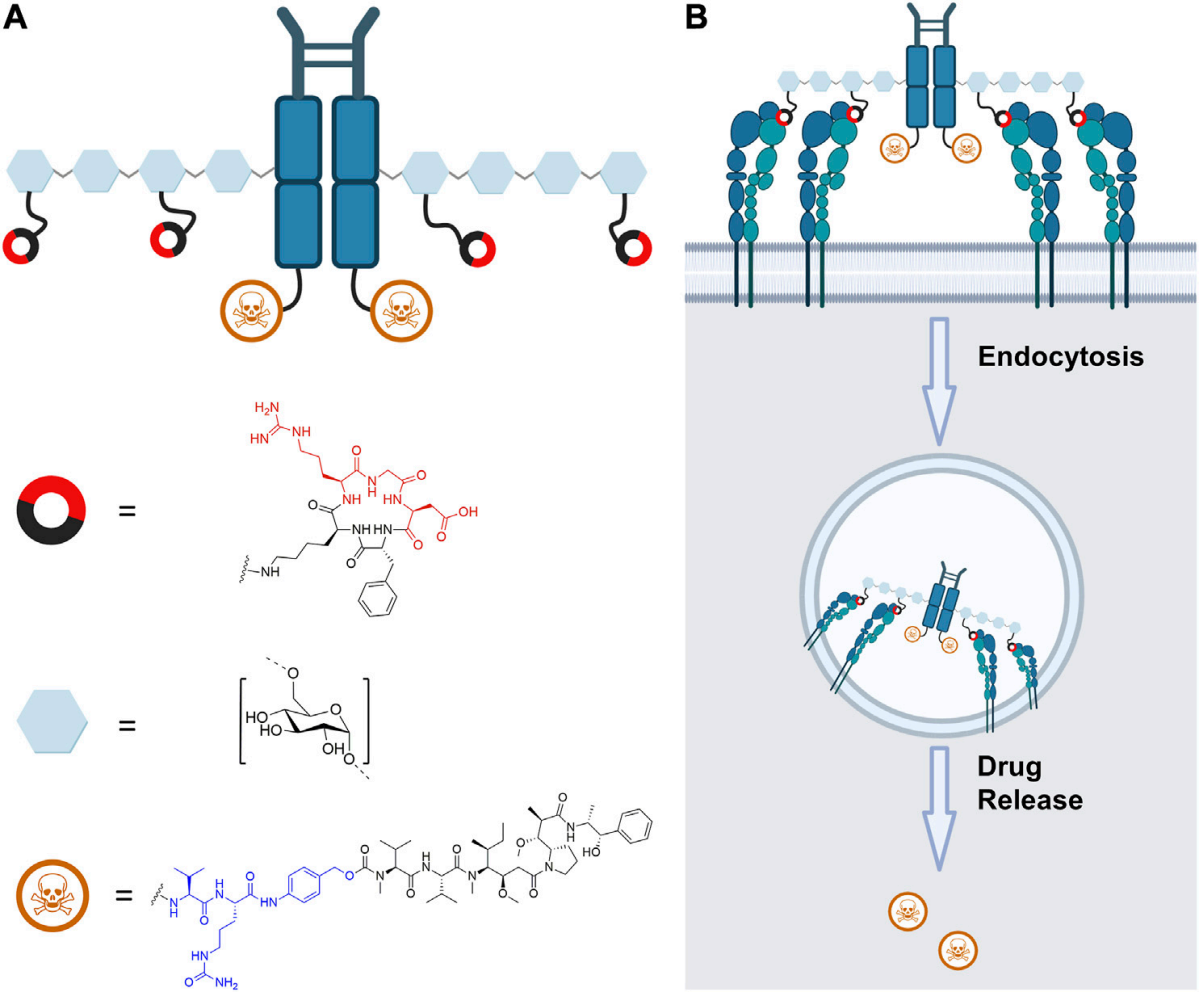
**(A)** Schematic depiction of human Fc functionalized with the cytotoxic agent MMAE (blue: Val-Cit-PAB linker), dextran, and RGD (red: relevant amino acids for receptor binding) with their respective structure. **(B)** Intended pathway of the RGD-decorated construct, whereby simultaneous binding of multiple integrins on the cellular surface promotes endocytosis. Cleavage of the self-immolative Val-Cit-PAB linker by endosomal cathepsin B leads to release of free MMAE. The figure was created with biorender.com.

Herein, we report the development and evaluation of a novel platform for the multimeric presentation of RGD ligands for targeted drug delivery relying on a dextran-Fc scaffold (Figure 1A). Dextran is a flexible polysaccharide comprised of *α*-(1–6)–linked d-glucose units with a low degree of branching (van Witteloostuijn et al., 2016), which can be modified to allow for the covalent attachment of cargo molecules. This polymer has been already used for the RGD-mediated delivery of doxorubicin and bortezomib (Li et al., 2020). Our group has recently reported dextran as a multimerization scaffold for novel ADCs (Schneider et al., 2019a), death receptor 5-targeting peptide (Schneider et al., 2019b), and cell-penetrating peptide L17E (Becker et al., 2021). Analogous to our previous work (Schneider et al., 2019b), a human Fc was chosen as a centerpiece of the construct, which serves as a scaffold for enzyme-mediated site-specific conjugation and, furthermore, enables purification by affinity chromatography. We chose the tubulin inhibitor MMAE as a potent cytotoxic agent (Chen et al., 2017), which was additionally equipped with a lysosomal cleavable and self-immolative valine-citrulline-PAB linker to enable release after endocytosis (Figure 1B) (Dubowchik et al., 2002).

## Materials and Methods

### Materials

10 kDa dextran from *Leuconostoc mesenteroides* (M_w_ = 9,000–11,000 g/mol, minimal α-(1-3) branching (5%)) was purchased from Sigma-Aldrich (St. Louis, United States). GGG-Val-Cit-PAB-MMAE was kindly provided by Merck KGaA (Darmstadt, Germany). Amino acids for solid-phase peptide synthesis (SPPS) and 2-(1*H*-benzotriazol-1-yl)-1,1,3,3-tetramethyluronium hexafluorophosphate (HBTU) were purchased from Iris Biotech (Marktredwitz, Germany). *N*,*N*-dimethylformamide (DMF), trifluoroacetic acid (TFA), piperidine, and *N*-ethyl-*N*-(propan-2-yl)propan-2-amine (DIEA) SPPS grade were purchased from Carl Roth (Karlsruhe, Germany). All other reagents were of analytical grade and were used without further purification. 3-(4,5-Dimethylthiazol-2-yl)-5-(3-carboxymethoxyphenyl)-2-(4-sulfophenyl)-2H-tetrazolium, inner salt (MTS) CellTiter96® AQueous One Solution cell viability assay was purchased from Promega (Madison, United States). IgG Fc goat anti-human, PE, eBioscience^TM^ for immune staining was purchased from Fisher Scientific (Hampton, United States).

### NMR Spectroscopy

NMR measurements were performed on a 300 MHz Avance II or 300 MHz Avance III spectrometer (Bruker BioSpin GmbH, Rheinstetten, Germany). Samples were dissolved in deuterium oxide or CDCl_3_.

### Mass Spectrometry

Mass spectra were recorded on a LCMS-2020 electrospray ionization (ESI) mass spectrometer from Shimadzu (Kyoto, Japan). The system was equipped with a Phenomenex (Aschaffenburg, Germany) Synergi 4u Fusion-RP 80 C18 (250 mm × 4.6 mm, 2 μm, 80 Å) column. The eluent system consisted of eluent A: 0.1% (v/v) aq. formic acid (FA) (LC-MS grade, Fisher Scientific), and eluent B: acetonitrile (ACN) containing 0.1% (v/v) FA (LC-MS grade) at a flow rate of 0.7 ml min^−1^.

### HPLC

Reversed-phase (RP) HPLC measurements were conducted on an Agilent Infinity 1100 device (Agilent, Santa Clara, United States) equipped with an Agilent Eclipse Plus RP column (C18, 3.5 µm, 100 mm × 4.6 mm, 95 Å) at a flow rate of 0.6 ml min^−1^. Alternatively, an Agilent Infinity 1260 device equipped with an Interchim (Montluçon, France) Uptisphere Strategy (C18-HQ, 3 μm, 100 mm× 4.6 mm) column was used. The eluent system consisted of eluent A: 0.1% (v/v) aq. TFA, and eluent B: 90% (v/v) aq. ACN containing 0.1% (v/v) TFA.

Product purities were estimated upon the percentage of peak area under the curves at 220 nm wavelength using the Agilent OpenLab Software.

Hydrophobic interaction chromatography (HIC) was performed on an Agilent Infinity 1260 device equipped with a Tosoh Bioscience GmbH (Griesheim, Germany) TSK Butyl-NPR column (2.5 µm, 35 mm × 4.6 mm). The eluent system consisted of eluent A: 1.5 M (NH_4_)_2_SO_4_ in 20 mM Tris pH 7.5, and eluent B: 20 mM Tris pH 7.5. Analysis was performed applying a 35 min gradient at a flow rate of 0.9 ml min^−1^.

Peptide purification was conducted on an Interchim (Montluçon, France) Puriflash 4250 semipreparative HPLC with an Interchim Uptisphere Strategy (C18-HQ, 5 μm, 250 mm × 21.2 mm) column at a flow rate of 18 ml min^−1^. The eluent system consisted of eluent A: 0.1% (v/v) aq. TFA, and eluent B: 90% (v/v) aq. ACN containing 0.1% (v/v) TFA.

### Synthesis of 4-Pentynoic Acid NHS Ester **17**

The synthesis was performed according to the literature (Pal and Koner, 2017). Thus, 4-pentynoic acid (200 mg, 2.04 mmol, 1 eq.) and N-hydroxysuccinimide (268 mg, 2.33 mmol, 1.15 eq.) were dissolved in 10 ml anhydrous dichloromethane (DCM). The reaction mixture was cooled with an ice bath, and 1-ethyl-3-(3-dimethylaminopropyl)carbodiimide hydrochloride (EDC·HCl) (780 mg, 4.07 mmol, 2 eq.) was added in portions. The reaction mixture was stirred for a further 2 h, filtered, and evaporated under reduced pressure. The residue was dissolved in ethyl acetate, washed thrice with saturated aqueous NaHCO_3_ and thrice with brine, and subsequently dried over Na_2_SO_4_. The organic phase was evaporated, and the residue was dissolved in ACN and precipitated by the addition of water. The suspension was freeze-dried, yielding 382 mg (1.96 mmol, 96.1%) as a colorless solid. ^1^H NMR (300 MHz, CDCl_3_) δ = 2.90–2.82 (m, 2H), 2.81 (s, 4H), 2.64–2.54 (m, 2H), and 2.03 (t, J = 2.6 Hz, 1H) ppm and ^13^C NMR (75 MHz, CDCl_3_) δ = 171.7, 168.9, 82.2, 70.9, 31.2, 26.4, and 14.7 ppm.

### Synthesis of *H*-D(OtBu)fK(Boc)R(Pbf)G-*OH* **11** and *H*-D(OtBu)fK(Boc)R(Pbf)A-*OH* **12**

Synthesis was performed as described in the literature (Dai et al., 2000) with some modifications. Thus, 2-chlorotrityl chloride resin (2-CTC resin, 0.75 mmol, loading: 1.59 mmol/g) was loaded with 4 eq. Fmoc-Gly-OH and Fmoc-Ala-OH, respectively, in DCM in the presence of 8 eq. DIEA for 2 h. Then the supernatant was drained and the resin was washed thoroughly with DMF. Fluorenylmethoxycarbonyl (Fmoc)-protecting group was removed with 20% (v/v) piperidine in DMF for 5 and 10 min. After thorough washing with DMF, the subsequent building blocks were coupled using 4 eq. Fmoc-protected amino acid, 3.95 eq. HBTU, and 8 eq. DIEA for 1 h in DMF. Fmoc-f-OH was coupled using 3 eq. amino acid, 2.95 eq. HBTU, and 6 eq. DIEA. After final Fmoc deprotection, the peptides were cleaved from resin under mild conditions using the mixture of acetic acid:DCM:methanol (5:4:1, v/v/v) for 2 h. Cleavage cocktail was removed under reduced pressure and the residue was dissolved in ACN, followed by precipitation by the addition of water. Freeze-drying of the suspension yielded 615 mg (0.60 mmol, 79.5%) *H*-D(OtBu)fK(Boc)R(Pbf)G-*OH* **11** and 664 mg (0.64 mmol, 84.8%) *H*-D(OtBu)fK(Boc)R(Pbf)A-*OH* **12** which were used without further purification.

*H*-D(OtBu)fK(Boc)R(Pbf)G-*OH* **11**: HPLC: t_R_ = 12.525 min (30–100% eluent B; 20 min gradient, purity = 79.2%); ESI-MS: m/z_calc._ = 1,030.53 [M+H]^+^ m/z_obs._ = 1,030.67, m/z_calc._ = 1,028.51 [M-H]^−^ m/z_obs._ = 1,028.47.

*H*-D(OtBu)fK(Boc)R(Pbf)A-*OH* **12**: HPLC: t_R_ = 12.635 min (30–100% eluent B; 20 min gradient, purity = 90.6%); ESI-MS: m/z_calc._ = 1,044.54 [M+H]^+^ m/z_obs._ = 1044.67, m/z_calc._ = 1042.53 [M-H]^−^ m/z_obs._ = 1,042.57.

### Synthesis of *Cyclo*[D(OtBu)fK(Boc)R(Pbf)G] **13** and *Cyclo*[D(OtBu)fK(Boc)R(Pbf)A] **14**

Cyclization of linear protected peptides **11** and **12** was performed in a modified procedure adapted from literature (Fernandez-Llamazares et al., 2013). Thus, 350 mg **11** and **12** (0.34 mmol, 1 eq.), respectively, were dissolved in DCM (1 mM), followed by the addition of 4-dimethylaminopyridine (4-DMAP) (83 mg, 0.68 mmol for **11**, 82 mg, 0.68 mmol for **12**, 2 eq.) and EDC·HCl (651 mg, 3.40 mmol for **11**, 643 mg, 3.35 mmol for **12**, 10 eq.). The reaction mixtures were stirred overnight at ambient temperature, and the solutions were concentrated *in vacuo*. The organic phases were extracted twice with 2% (v/v) formic acid, two times with saturated aqueous NaHCO_3_, and thrice with brine and dried over MgSO_4_. Evaporation of solvent, precipitation in ACN:H_2_O, and subsequent freeze-drying yielded 177 mg **13** (0.18 mmol, 51.6%) and 214 mg **14** (0.21 mmol, 62.2%).

*cyclo*[D(OtBu)fK(Boc)R(Pbf)G] **13**: HPLC: t_R_ = 16.784 min (30–100% eluent B; 20 min gradient, purity = 57.8%); ESI-MS: m/z_calc._ = 1,012.52 [M+H]^+^ m/z_obs._ = 1,012.67.

*cyclo*[D(OtBu)fK(Boc)R(Pbf)A] **14**: HPLC: t_R_ = 16.903 min (30–100% eluent B; 20 min gradient, purity = 68.6%); ESI-MS: m/z_calc._ = 1,026.53 [M+H]^+^ m/z_obs._ = 1,026.77.

### Synthesis of *Cyclo*[RGDfK] **15** and *Cyclo*[RADfK] **16**

Side-chain deprotection of the cyclized protected peptide **13** (177 mg, 0.18 mmol) or **14** (214 mg, 0.21 mmol) was achieved by stirring with the TFA:DCM (1:1, v/v) mixture at ambient temperature for 2 h. The deprotected peptides were precipitated in cold diethyl ether, centrifuged, and washed thrice with diethyl ether. The residues were dried, dissolved in water, and freeze-dried. Purification of the crude products was performed by semipreparative HPLC (0–45% eluent B, 20 min gradient). Pooling of the product fractions yielded 24 mg **15** (0.04 mmol, 22.1%). For **16**, two product fractions with 24 mg (0.04 mmol, 18.5%) and 47 mg (0.08 mmol, 36.2%) were obtained. MS analysis showed for both fractions identical m/z signals, which suggests the presence of stereoisomers. Hence, both fractions were pooled for the following reaction.

*cyclo*[RGDfK] **15**: HPLC: t_R_ = 13.295 min (0–40% eluent B; 20 min gradient, purity = 100%); ESI-MS: m/z_calc._ = 604.32 [M+H]^+^ m/z_obs._ = 604.37, m/z_calc._ = 602.31 [M-H]^−^ m/z_obs._ = 602.27.

*cyclo*[RADfK] **16**: HPLC: t_R_ = 13.156 min^#^, 13.922 min^*^ (0–40% eluent B; 20 min gradient, purity = 91.6%^#^, 80.6%^*^); ESI-MS: m/z_calc._ = 618.34 [M+H]^+^ m/z_obs._ = 618.48^#^, 618.38^*^, m/z_calc._ = 616.32 [M-H]^−^ m/z_obs._ = 616.38^#^, 616.38^*^; #,*: corresponding to the isomers.

### Synthesis of *Cyclo*[RGDfK(alkyne)] **18** and *Cyclo*[RADfK(alkyne)] **19**

Twenty-four milligrams of **15** (0.04 mmol, 1 eq.) or 70 mg of **16** (0.11 mmol, 1 eq.) and 4-pentynoic acid-NHS ester **17** (14 mg, 0.07 mmol for **15**, 40 mg, 0.20 mmol for **16**, 1.8 eq.) were dissolved in dry dimethyl sulfoxide (DMSO). Anhydrous DIEA (21 mg, 28 μl, 0.16 mmol for **15**, and 58 mg, 79 μl, 0.45 mmol for **16**, 4 eq.) was added and the reaction mixture was stirred overnight at 30°C, followed by freeze-drying. The crude products were purified by semipreparative HPLC (0–40% eluent B, 20 min gradient). Freeze-drying of the product fractions yielded 10 mg **18** (14.6 µmol, 36.7%) and 50 mg **19** (71.7 µmol, 63.5%).

*cyclo*[RGDfK(alkyne)] **18**: HPLC: t_R_ = 14.190 min (10–50% eluent B; 20 min gradient, purity = 94.9%); ESI-MS: m/z_calc._ = 684.35 [M+H]^+^ m/z_obs._ = 684.38, m/z_calc._ = 682.33 [M-H]^−^ m/z_obs._ = 682.38.

*cyclo*[RADfK(alkyne)] **19**: HPLC: t_R_ = 14.077 min^#^, 14.559 min^*^ (10–50% eluent B; 20 min gradient, purity = 35.9%^#^, 60.7%^*^); ESI-MS: m/z_calc._ = 698.36 [M+H]^+^ m/z_obs._ = 698.48, m/z_calc._ = 696.35 [M-H]^-^ m/z_obs._ = 696.35.

### Dextran Functionalization

Dextran functionalization was performed as previously reported (Schneider et al., 2019b). Analytical data of the final dextran derivative cadaverine–dextran–(N_3_)_15.6_ **9** are given in Supplementary Material.

### Protein Expression and Purification

Expression and purification of aglycosylated (N297) human Fc-LPETGG **1** were realized as previously reported (Schneider et al., 2019b). Briefly, the DNA sequence for the protein was cloned in a standard pEXPR vector for mammalian expression. Forty micrograms of the plasmid was mixed with 120 µg polyethylenimine (PEI) in a serum-free Expi293^TM^ expression medium (Thermo Fisher Scientific, Waltham, United States). The mixture was added dropwise to 2.5 × 10^6^ Expi293F^TM^ cells/ml in 30 ml Expi293^TM^ expression medium. After 24 h incubation under constant shaking, 0.5% (w/v) tryptone was added and the cells were incubated for further 5 days. Following this, the supernatant was diluted with an equal volume of protein A running buffer (20 mM sodium phosphate, pH 7) and purified by protein A affinity chromatography (HiTrap Protein A HP column, GE Healthcare, Chicago, United States). Elution of Fc **1** was performed with 100 mM citrate buffer pH 3. Product fractions were pooled, dialyzed against phosphate-buffered saline (PBS) pH 7.4, and concentrated using Amicon Ultra centrifugal filters (Merck Millipore, Burlington, United States).

Microbial transglutaminase (mTG, *Streptomyces mobaraensis*) was produced and activated with trypsin as described in the literature (Deweid et al., 2018).

Sortase A was expressed and purified according to the literature (Chen et al., 2011).

### Sortase A-Mediated Condensation

Enzyme-mediated conjugation of GGG-Val-Cit-PAB-MMAE **2** to Fc-LPETGG **1** was carried out in a modified variant from the literature (Beerli et al., 2015). Fc **1** was diluted to 1 mg ml^−1^ (18.8 µM, 1 eq.) in reaction buffer (50 mM Tris, 150 mM NaCl, 10 mM CaCl_2_, pH 7.5). MMAE derivative **2** was added (final concentration 188 μM, 10 eq.), followed by 0.1 eq. sortase A (1.88 µM). The reaction was incubated at 22°C for 90 min, and reaction control was realized by HIC and SDS-PAGE. Fc-MMAE conjugate **3** was isolated by Protein A HP SpinTrap (Prot A, GE Healthcare) columns and concentrated afterward with Amicon Ultra centrifugal filters.

### Transglutaminase-Mediated Transamidation

Conjugation of cadaverine–dextran–(N_3_)_15.6_ **9** to Fc-MMAE **3** or Fc **1** was performed according to the literature (Schneider et al., 2019b). Fc-MMAE **3** or Fc **1** was diluted to 1.33 mg ml^−1^ (25 μM, 1 eq.) in Tris buffer pH 8 (25 mM Tris, 150 mM NaCl). (N_3_)_16.5_–dextran–cadaverine **9** (final concentration 2 mM, 80 eq.) and mTG (final concentration 6.25 µM, 7.3 U ml^−1^, 0.25 eq.) were added and the reaction was incubated at 22°C for 24 h. Products **10** and **22**, respectively, were isolated using Prot A columns and remained on the columns for further modification. Small portions of the products were eluted and analyzed by SDS–PAGE.

### Copper(I)-Catalyzed Azide-Alkyne Cycloaddition on Fc-Dextran

CuAAC of alkyne-modified RGD or RAD to Fc-dextran was performed as described in the literature (Schneider et al., 2019b). Prot A-immobilized Fc-dextran-MMAE **10** or Fc-dextran **22** (1 eq.) in PBS was mixed with either alkyne-RGD **18** or alkyne-RAD **19** (2.5 eq. per N_3_). Fresh stocks of ascorbic acid (5 eq. per N_3_) and CuSO_4_·5H_2_O (2.5 eq. per N_3_) in water were mixed, incubated for 5 min, and added to the immobilized proteins. The reactions were incubated for 3 h at 30°C, followed by the removal of the solvent and washing of the immobilized proteins with PBS and subsequent elution. The buffer was exchanged to PBS and the products **20**, **21**, **23**, and **24** were concentrated with Amicon Ultra centrifugal filters. Reaction success was confirmed by SDS-PAGE.

### Cell Culture

Cells were incubated under standard conditions in a humidified incubator with 5% CO_2_ at 37°C. Expi293F^TM^ cells were incubated in serum-free Expi293^TM^ expression medium in an orbital shaker at 110 rpm and 8% CO_2_. U87MG cells were incubated in minimum essential medium (MEM) supplemented with 10% (v/v) fetal bovine serum (FBS).

### Cell Proliferation Assay

U87MG cells were seeded in 96-well plates with a density of 3.5 × 10^3^ cells and incubated for 7 h under standard conditions. Serial dilutions of samples (10× concentrated in PBS) were added to a final volume of 100 µl and the cells were incubated for a further 72 h. MTS (AQueous One Solution) was added and cell proliferation was measured using a Tecan Infinite F200 PRO (Männedorf, Switzerland). Samples in reference wells containing untreated cells were set to 100% viability.

### Cell Binding Assay

Trypsinized U87MG cells were washed twice with 0.1% (w/v) bovine serum albumin (BSA) in PBS. 2.5 × 10^5^ cells were transferred to a 96-well plate and incubated with the respective concentration of constructs in PBS + 0.1% BSA for 35 min on ice. The supernatant was removed and the cells were washed thrice with 0.1% BSA in PBS. Following this, the cells were incubated for 20 min on ice with fluorescently labeled IgG Fc goat anti-human, PE, eBioscience^TM^ (1/100 diluted in PBS + 0.1% BSA). Subsequently, cells were washed once with 0.1% BSA in PBS and analyzed by flow cytometry using a BD influx device (Becton Dickinson, Franklin Lakes, United States).

### Thermal Shift Assay

Thermal shift assays were performed as duplicates on a CFX96 device (Bio-Rad, Hercules, United States). Measurements were performed at protein concentrations of 0.1 mg ml^−1^ in PBS using SYPRO Orange (dilution 1:800) whereby temperature was increased in 0.5°C/30 s increments up to 99°C. Melting temperatures (T_m_) were derived from melting curves using the Bio-Rad software.

### Statistical Analysis

Statistical analysis was performed with GraphPad Prism version 8.0.1. Results are displayed as the mean ± standard error of the mean and are based on triplicates. Statistical significance was determined via a two-way ANOVA test (Bonferroni t-test). *p* values ≤ 0.05 were considered to be statistically significant.

## Results and Discussion

### Design and Synthesis

**FIGURE 2:**
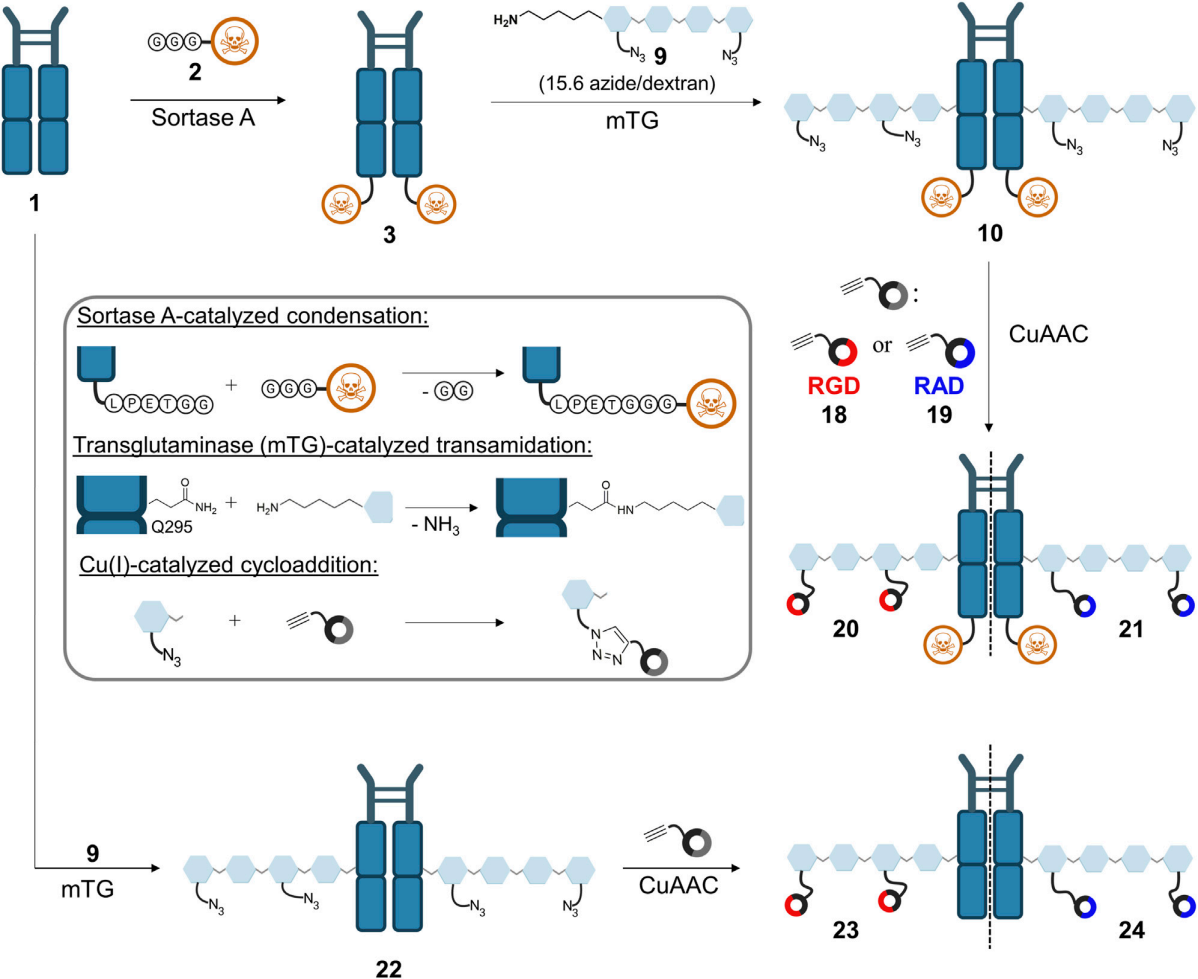
Synthetic approach toward the final constructs **20** and **21**, starting with sortase A-mediated conjugation of MMAE **2** to Fc **1**, followed by transglutaminase-catalyzed conjugation of azide-decorated dextran **9**. Construct **10** was further functionalized via CuAAC with either alkyne-decorated RGD **18** or RAD **19** to yield final constructs **20** and **21**. Alternatively, Fc **1** was directly functionalized with dextran (construct **22**) and further modified via CuAAC to obtain RGD-decorated **23** and RAD-decorated **24**. Figure was created with biorender.com.

**FIGURE 3:**
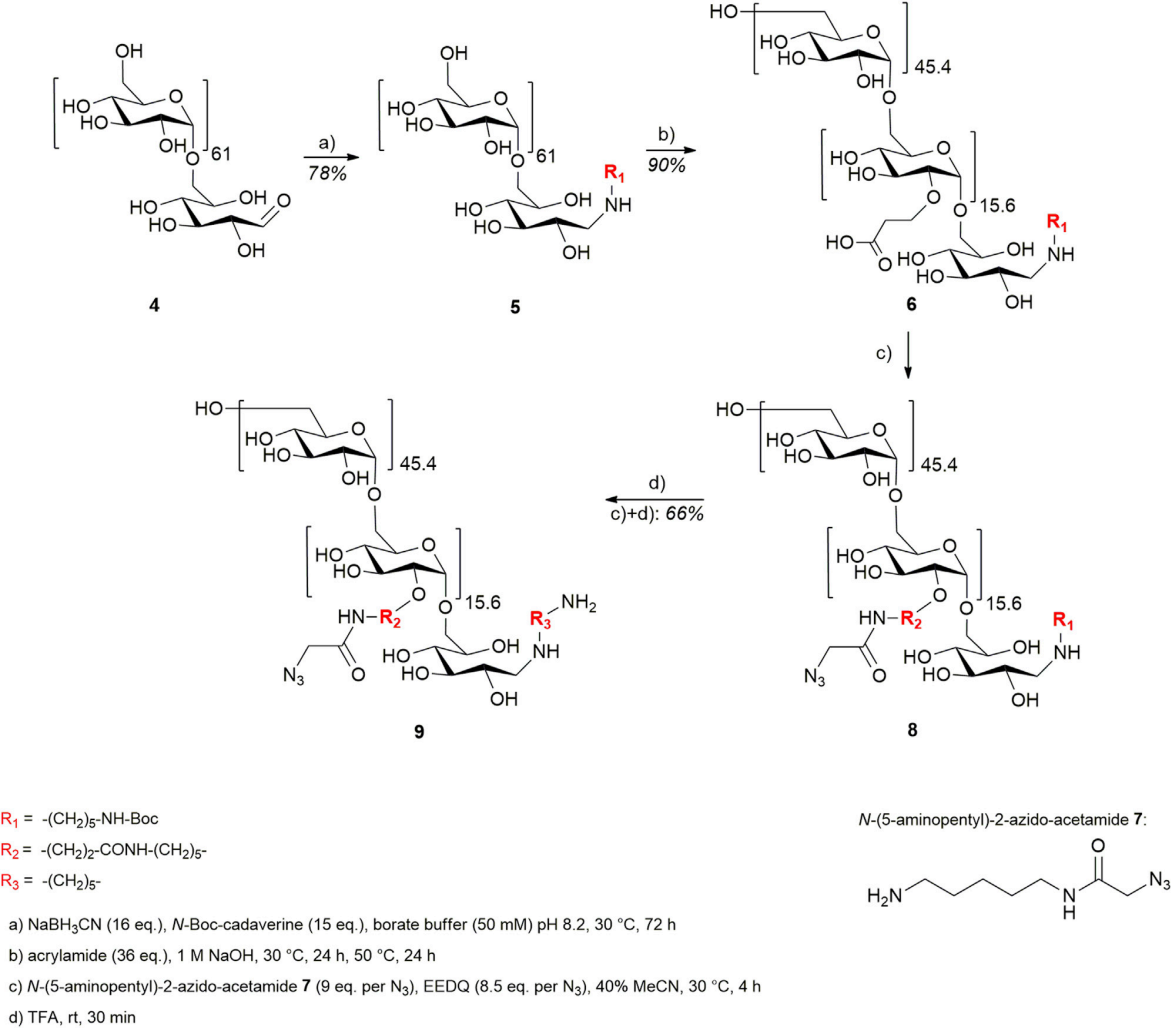
Synthetic approach toward cadaverine–dextran–(N_3_)_15.6_ **9**.

**FIGURE 4:**
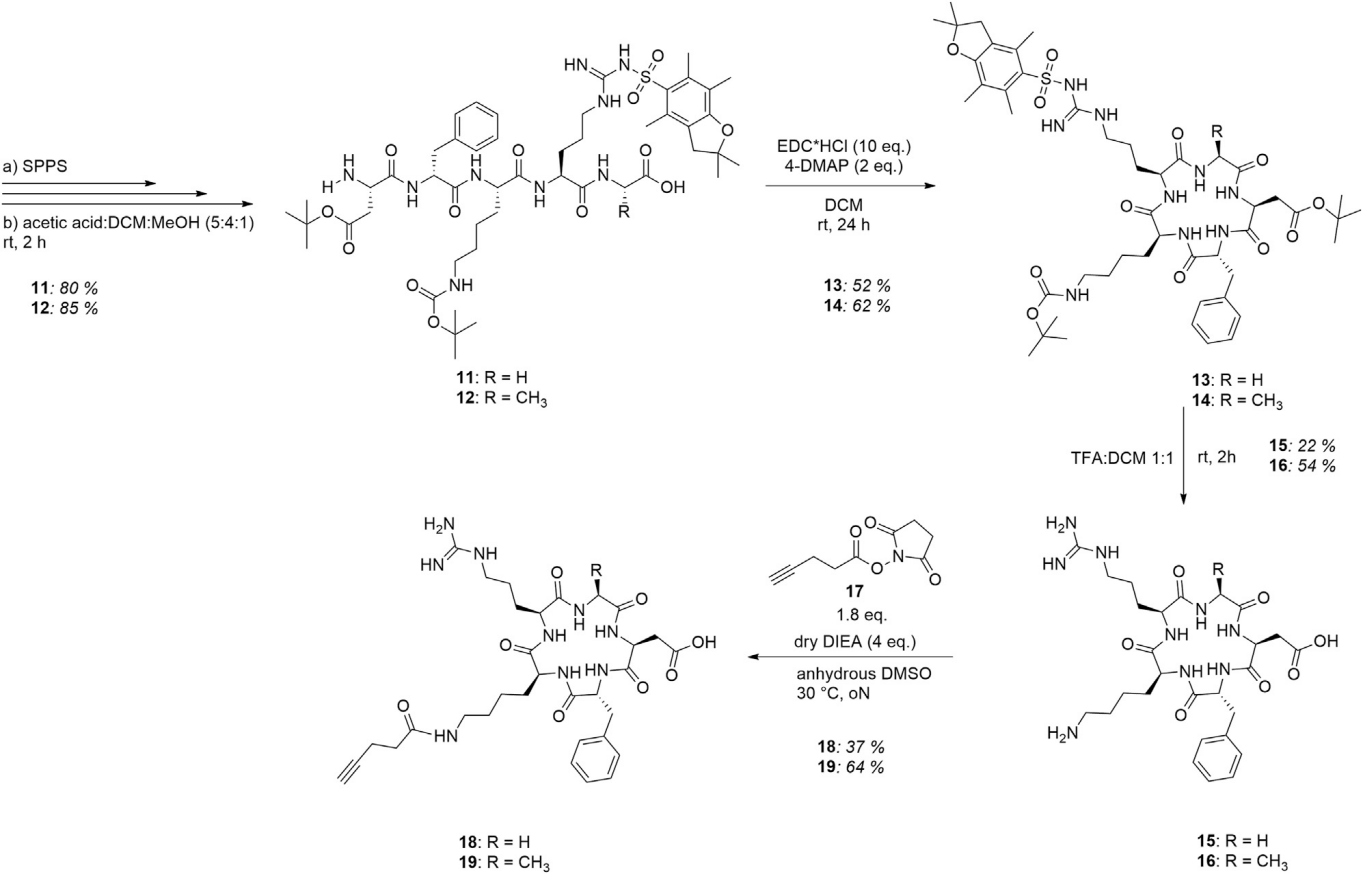
Synthesis route to alkyne-functionalized *cyclo*[RGDfK] **18** and *cyclo*[RADfK] **19**, respectively.

To ensure orthogonal and site-specific conjugation of both dextran and cytotoxic agent, two enzyme recognition sites were introduced into Fc **1**. For sortase A-mediated conjugation of GGG-PEG_3_-Val-Cit-PAB-MMAE **2**, a C-terminal LPETGG extension was incorporated genetically (Macarrón Palacios et al., 2020). Furthermore, Fc was produced as an aglycosylated variant N297A which enables coupling of cadaverine-functionalized dextran to Q295 by microbial transglutaminase (Schneider et al., 2019b). The modification of Fc started with sortase A-catalyzed conjugation of MMAE **2** (Figure 2), and product **3** was isolated using protein A affinity chromatography. Fc-MMAE **3** was analyzed by HIC, whereby the drug-to-protein ratio was 1.6 according to the integrals of the corresponding peaks (Supplementary Figure S28). In the next step, dextran **9** was conjugated to Fc-MMAE **3** using transglutaminase (Figure 2), an enzyme catalyzing the formation of isopeptide bonds between suited glutamine residues and amine counterparts. Therefore, 10 kDa (corresponds to 62 glucose units on average) dextran **4** was equipped with a Boc-protected cadaverine moiety via reductive amination (compound **5**) and was further functionalized with 15.6 carboxyethyl groups at C2 position on average (compound **6**) (Figure 3). EEDQ-mediated conjugation of amine-bearing linker **7** to the carboxy groups yielded azide-functionalized dextran **8**. Finally, acidic cleavage of the Boc-protecting group at the reducing end resulted in dextran **9**. After mTG-mediated conjugation of azide-bearing dextran **9** to Fc-MMAE **3**, product **10** was isolated by protein A affinity chromatography and remained immobilized for the subsequent modification via copper “click” reaction. Therefore, alkyne-modified *cyclo*[RGDfK] **18** and, additionally, *cyclo*[RADfK] **19** as negative control were required (Figure 4), which were obtained by solid-phase peptide synthesis using the standard Fmoc strategy according to the literature (Dai et al., 2000). After mild cleavage to conserve the side chain-protecting groups, the linear peptides **11** and **12** were head-to-tail cyclized at low concentrations to minimize side reactions. Subsequent deprotection of cyclic peptides **13** and **14** under acidic conditions yielded *cyclo*[RGDfK] **15** and *cyclo*[RADfK] **16**. Interestingly, the HPLC analysis of crude *cyclo*[RADfK] **16** (Supplementary Figure S15) displayed two product peaks at approximately equal ratios and identical m/z signals. This indicates the formation of stereoisomers by the racemization of the *C*-terminal amino acid as has been reported for head-to-tail cyclization of peptides (Davies, 2003). This assumption is supported by the fact that this did not take place for the synthesis of *cyclo*[RGDfK] **15** (Supplementary Figure S12), which is based on a *C*-terminal glycine. However, it remains unclear why no chromatographic separation of the isomers did occur for the side chain-protected precursor **14**. An alkyne moiety was introduced by coupling of 4-pentynoic acid NHS ester **17** to the respective lysine side chains. Conjugation of alkyne-functionalized *cyclo*[RGDfK] **18** and *cyclo*[RADfK] **19**, respectively, to protein A-immobilized Fc-MMAE-dextran **10** was achieved by copper “click” reaction (Figure 2) which yielded the final constructs **20** (RGD-decorated) and **21** (RAD-decorated). In addition to the MMAE-functionalized constructs **20** and **21**, Fc-dextran-containing RGD (construct **23**) and RAD (construct **24**), respectively, lacking a cytotoxic warhead were synthesized as controls. To that end, Fc **1** was functionalized with dextran **9** via transglutaminase-mediated transamidation yielding Fc-dextran **22** (Figure 2), which was further decorated with alkyne-bearing *cyclo*[RGDfK] **18** and *cyclo*[RADfK] **19** (Figure 2), respectively, to obtain constructs **23** and **24**.

### Biology

**FIGURE 5:**
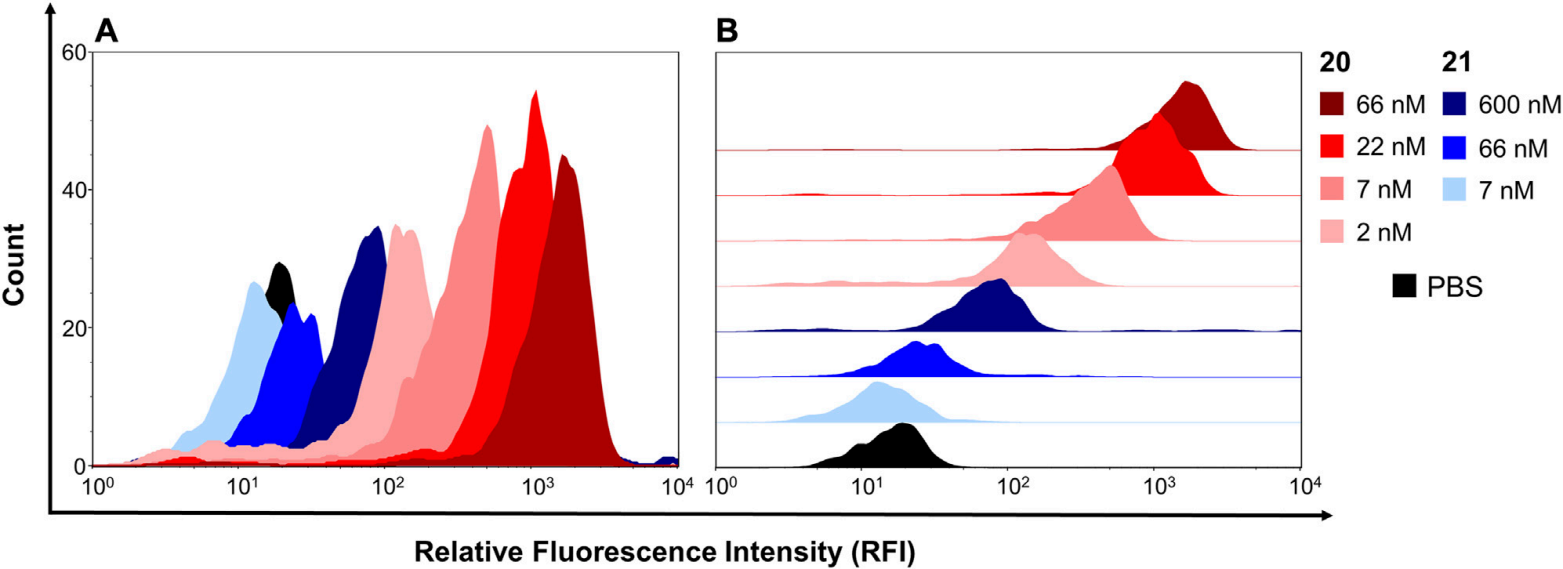
Binding of construct **20** and construct **21** to U87MG cells, displayed as histogram **(A)** and half offset **(B)**.

**FIGURE 6:**
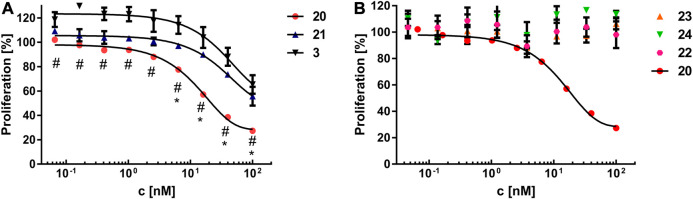
**(A)** Cell proliferation assay of MMAE-functionalized constructs **20**, **21**, and **3** on U87MG cells. Results are displayed as mean ± standard error of the mean and are based on triplicates. Significant differences (*p* ≤ 0.05) between **20** and **21** or **20** and **3** are depicted as (*) and (#), respectively. **(B)** Cell proliferation assay of constructs **20**, **22**, **23**, and **24** on U87MG cells. Results are shown as the mean ± standard error of the mean and are based on triplicates.

In order to assess, whether the modifications had an impact on the stability of the constructs, the melting properties of construct **20** were compared to parent Fc **1**. Thermal shift assay (Supplementary Figures S38, S39) revealed only negligible differences between construct **20** (T_m_ = 64.75 ± 0.25°C) and nonfunctionalized Fc **1** (T_m_ = 66.00 ± 0.50°C). Next, the binding properties of the constructs **20** and **21** on U87MG cells, which display high levels of αvβ3 on their surface (Benedetto et al., 2006), were investigated. To that end, trypsinized U87MG cells were incubated with either **20** or **21** at different concentrations, followed by labeling of the Fc part with a detection antibody and subsequent flow cytometry. RGD-functionalized construct **20** showed higher binding capabilities to U87MG cells compared to RAD-decorated construct **21** (Figure 5). These findings clearly demonstrate that specific interactions of RGD-containing construct **20** with the cellular surface of U87MG cells play a pivotal role for binding, since RAD-modified **21**, which has alanine instead of glycine in the cyclic peptide, shows negligible binding. The binding of RGD-containing construct **20** revealed an apparent dissociation constant K_D_ of 33 nM, whereas no K_D_ value for the RAD-decorated construct **21** could be determined since no sigmoidal binding curve—up to a concentration of 600 nM—was observed (Supplementary Figure S40). Encouraged by these results, we investigated the impact of RGD- and RAD-modified constructs **20** and **21**, respectively, as well as Fc-MMAE **3**, on the proliferation of U87MG cells. Cells were treated with compounds at different concentrations for 3 days, after which the proliferation was quantified in an MTS assay. RGD-decorated construct **20** displayed higher antiproliferative effects (EC_50_ = 16.15 nM) compared to the RAD-decorated counterpart **21** (EC_50_ > 100 nM, Figure 6A). This observation strongly suggests an integrin-dependent uptake of construct **20** which results in higher intracellular concentrations of MMAE and, ultimately, higher cytotoxicity. The antiproliferative activity of construct **21** (EC_50_ > 100 nM) was found similar to Fc-MMAE **3**, implying nonspecific activity of RAD-decorated **21**. This could be caused by either nonspecific uptake or hydrolysis of the linkage between Fc and cytotoxic agent, which is membrane-permeable in free form, within the incubation time. In comparison with a solitary RGD-containing peptide connected to MMAE by a lysosomally cleavable Val-Ala linker, construct **20** displayed higher cytotoxic activity in U87MG cells than its monomeric counterpart (IC_50_ = 39 nM) (Raposo Moreira Dias et al., 2019). To investigate whether multimerized RGD without cytotoxic payload exhibits antiproliferative effect by receptor clustering, cytotoxicity of construct **23** was assessed and compared to RAD-decorated construct **24** (Figure 6B). Both constructs **23** and **24**, respectively, display no cytotoxicity—up to a concentration of 600 nM—highlighting the necessity of a cytotoxic payload for RGD-directed killing of tumor cells.

## Conclusion and Outlook

The combination of unspecific cytotoxic agents with targeting moieties offers a promising strategy towards targeted antitumor therapy. Peptides incorporating the RGD-motif have proven their efficacy as integrin binders in numerous publications, whereby their potency was even further enhanced by multimerization. Herein, we present the design, synthesis, and biological evaluation of a novel scaffold bearing multiple RGD peptides in combination with a potent cytotoxin. Based on a nonglycosylated Fc as a centerpiece, endosomal-cleavable MMAE as cytotoxic agent and dextran as multimerization site were introduced by enzyme-mediated site-specific reactions. Multiple attachments of RGD peptides were achieved by copper “click” reaction resulting in final construct **20**. These modifications had little impact on the stability. Indeed, negligible differences in melting temperatures between construct **20** and parent Fc **1** were observed. RGD-containing construct **20** showed superior binding of αvβ3-expressing U87MG cells compared to the negative control, that is, RAD-decorated construct **21**. These properties also translated into their antiproliferative activity, and construct **20** displayed significantly higher cytotoxicity than control **21**. In these constructs, the binding is not mediated by the antibody part, but by dextran decorated with a high number of cyclic peptidic integrin binders. Thus, this format enables to transcribe small binders into an antibody-like format. The observed potency of 16.15 nM is comparable to a cystine knot peptide genetically fused to Fc which binds integrins containing an αv subunit with high affinity and was equipped with 1.9 MMAF (EC_50_ = 9.2 nM) (Currier et al., 2016). We recently showed that multiple MMAE conjugations to dextran result in higher cytotoxicity compared to single coupling as used in this study (Schneider et al., 2019a). Further work will reveal whether the usage of a dual modified Fc with a variable number of multiple copies of the integrin-binding peptides and of the cytotoxin both installed separately on dextran result in tunable cell-specific cytotoxicity. Already in the present setting, our results demonstrate the general capability of our design to provoke receptor-mediated endocytosis upon binding to the cellular surface, followed by endosomal cleavage of the linkage between Fc-dextran and MMAE and its subsequent release. In conclusion, our approach opens new avenues for the development of tailor-made multimeric molecular hybrids with antitumor potential.

## Data Availability

The raw data supporting the conclusions of this article will be made available by the authors, without undue reservation, to any qualified researcher.

## References

[B1] Aumailley M. Gurrath M. Müller G. Calvete J. Timpl R. Kessler H. (1991). Arg-Gly-Asp Constrained Within Cyclic Pentapoptides Strong and Selective Inhibitors of Cell Adhesion to Vitronectin and Laminin Fragment P1. FEBS Lett. 291, 50–54. 10.1016/0014-5793(91)81101-D 1718779

[B2] Becker B. Englert S. Schneider H. Yanakieva D. Hofmann S. Dombrowsky C. (2021). Multivalent Dextran Hybrids for Efficient Cytosolic Delivery of Biomolecular Cargoes. J. Pep. Sci. 27, e3298. 10.1002/psc.3298 33458922

[B3] Beerli R. R. Hell T. Merkel A. S. Grawunder U. (2015). Sortase Enzyme-Mediated Generation of Site-Specifically Conjugated Antibody Drug Conjugates with High *In Vitro* and *In Vivo* Potency. PLoS One 10, e0131177. 10.1371/journal.pone.0131177 26132162 PMC4488448

[B4] Benedetto S. Pulito R. Crich S. G. Tarone G. Aime S. Silengo L. (2006). Quantification of the Expression Level of Integrin Receptor αvβ3 in Cell Lines and MR Imaging with Antibody-Coated Iron Oxide Particles. Magn. Reson. Med. 56, 711–716. 10.1002/mrm.21023 16958071

[B5] Borsari C. Trader D. J. Tait A. Costi M. P. (2020). Designing Chimeric Molecules for Drug Discovery by Leveraging Chemical Biology. J. Med. Chem. 63, 1908–1928. 10.1021/acs.jmedchem.9b01456 32023055 PMC7997565

[B6] Burkhart D. J. Kalet B. T. Coleman M. P. Post G. C. Koch T. H. (2004). Doxorubicin-Formaldehyde Conjugates Targeting Alphavbeta3 Integrin. Mol. Cancer Ther. 3, 1593–1604. 15634653

[B7] Chen H. Lin Z. Arnst K. Miller D. Li W. (2017). Tubulin Inhibitor-Based Antibody-Drug Conjugates for Cancer Therapy. Molecules 22, 1281. 10.3390/molecules22081281 PMC6152078 28763044

[B8] Chen H. Niu G. Wu H. Chen X. (2016). Clinical Application of Radiolabeled RGD Peptides for PET Imaging of Integrin αvβ3. Theranostics 6, 78–92. 10.7150/thno.13242 26722375 PMC4679356

[B9] Chen I. Dorr B. M. Liu D. R. (2011). A General Strategy for the Evolution of Bond-Forming Enzymes Using Yeast Display. Proc. Natl. Acad. Sci. 108, 11399–11404. 10.1073/pnas.1101046108 21697512 PMC3136257

[B10] Colombo R. Mingozzi M. Belvisi L. Arosio D. Piarulli U. Carenini N. (2012). Synthesis and Biological Evaluation (*In Vitro* and *In Vivo*) of Cyclic Arginine-Glycine-Aspartate (RGD) Peptidomimetic-Paclitaxel Conjugates Targeting Integrin αVβ3. J. Med. Chem. 55, 10460–10474. 10.1021/jm301058f 23140358

[B11] Currier N. V. Ackerman S. E. Kintzing J. R. Chen R. Filsinger Interrante M. Steiner A. (2016). Targeted Drug Delivery With an Integrin-Binding Knottin-Fc-MMAF Conjugate Produced by Cell-Free Protein Synthesis. Mol. Cancer Ther. 15, 1291–1300. 10.1158/1535-7163.MCT-15-0881 27197305

[B12] Dai X. Su Z. Liu J. O. (2000). An Improved Synthesis of a Selective αvβ3-integrin Antagonist Cyclo(-RGDfK-). Tetrahedron Lett. 41, 6295–6298. 10.1016/S0040-4039(00)01060-1

[B13] Davies J. S. (2003). The Cyclization of Peptides and Depsipeptides. J. Pept. Sci. 9, 471–501. 10.1002/psc.491 12952390

[B14] Dechantsreiter M. A. Planker E. Mathä B. Lohof E. Hölzemann G. Jonczyk A. (1999). N-Methylated Cyclic RGD Peptides as Highly Active and Selective αVβ3 Integrin Antagonists. J. Med. Chem. 42, 3033–3040. 10.1021/jm970832g 10447947

[B15] Desgrosellier J. S. Cheresh D. A. (2010). Integrins in Cancer: Biological Implications and Therapeutic Opportunities. Nat. Rev. Cancer 10, 9–22. 10.1038/nrc2748 20029421 PMC4383089

[B16] Deweid L. Neureiter L. Englert S. Schneider H. Deweid J. Yanakieva D. (2018). Directed Evolution of a Bond‐Forming Enzyme: Ultrahigh‐Throughput Screening of Microbial Transglutaminase Using Yeast Surface Display. Chem. Eur. J. 24, 15195–15200. 10.1002/chem.201803485 30047596

[B17] Dubowchik G. M. Firestone R. A. Padilla L. Willner D. Hofstead S. J. Mosure K. (2002). Cathepsin B-Labile Dipeptide Linkers for Lysosomal Release of Doxorubicin from Internalizing Immunoconjugates: Model Studies of Enzymatic Drug Release and Antigen-Specific *In Vitro* Anticancer Activity. Bioconjug. Chem. 13, 855–869. 10.1021/bc025536j 12121142

[B44] Fernandez-Llamazares A. I. Garcia J. Soto-Cerrato V. Perez-Tomas R. Spengler J. Albericio F. (2013). N-Triethylene Glycol (N-TEG) As a Surrogate for the N-Methyl Group: Application to Sansalvamide A Peptide Analogs. Chem. Commun. 49, 6430–6432. 10.1039/C3CC41788C 23752923

[B18] Humphries J. D. Byron A. Humphries M. J. (2006). Integrin Ligands at a Glance. J. Cell Sci. 119, 3901–3903. 10.1242/jcs.03098 16988024 PMC3380273

[B19] Kemker I. Feiner R. C. Müller K. M. Sewald N. (2020). Size‐Dependent Cellular Uptake of RGD Peptides. ChemBioChem 21, 496–499. 10.1002/cbic.201900512 31478590 PMC7064889

[B20] Khongorzul P. Ling C. J. Khan F. U. Ihsan A. U. Zhang J. (2020). Antibody-Drug Conjugates: A Comprehensive Review. Mol. Cancer Res. 18, 3–19. 10.1158/1541-7786.MCR-19-0582 31659006

[B21] Kok R. J. Schraa A. J. Bos E. J. Moorlag H. E. Ásgeirsdóttir S. A. Everts M. (2002). Preparation and Functional Evaluation of RGD-Modified Proteins as αvβ3Integrin Directed Therapeutics. Bioconjug. Chem. 13, 128–135. 10.1021/bc015561+ 11792188

[B22] Komazawa H. Saiki I. Igarashi Y. Azuma I. Kojima M. Orikasa A. (1993). Inhibition of Tumor Metastasis by a Synthetic Polymer Containing a Cell-Adhesive RGDS Peptide. J. Bioact. Compat. Polym. 8, 258–274. 10.1177/088391159300800305

[B23] Krall N. Scheuermann J. Neri D. (2013). Small Targeted Cytotoxics: Current State and Promises from DNA-Encoded Chemical Libraries. Angew. Chem. Int. Ed. 52, 1384–1402. 10.1002/anie.201204631 23296451

[B24] Li D. Su T. Ma L. Yin F. Xu W. Ding J. (2020). Dual-Acidity-labile Polysaccharide-di-Drugs Conjugate for Targeted Cancer Chemotherapy. Eur. J. Med. Chem. 199, 112367. 10.1016/j.ejmech.2020.112367 32474350

[B25] López Rivas P. Müller C. Breunig C. Hechler T. Pahl A. Arosio D. (2019). β-Glucuronidase Triggers Extracellular MMAE Release From an Integrin-Targeted Conjugate. Org. Biomol. Chem. 17, 4705–4710. 10.1039/C9OB00617F 31020985

[B26] Macarrón Palacios A. Grzeschik J. Deweid L. Krah S. Zielonka S. Rösner T. (2020). Specific Targeting of Lymphoma Cells Using Semisynthetic Anti-Idiotype Shark Antibodies. Front. Immunol. 11, 560244. 10.3389/fimmu.2020.560244 33324393 PMC7726437

[B27] Marchini M. Mingozzi M. Colombo R. Guzzetti I. Belvisi L. Vasile F. (2012). Cyclic RGD Peptidomimetics Containing Bifunctional Diketopiperazine Scaffolds as New Potent Integrin Ligands. Chem. Eur. J. 18, 6195–6207. 10.1002/chem.201200457 22517378

[B28] Nahrwold M. Weiß C. Bogner T. Mertink F. Conradi J. Sammet B. (2013). Conjugates of Modified Cryptophycins and RGD-Peptides Enter Target Cells by Endocytosis. J. Med. Chem. 56, 1853–1864. 10.1021/jm301346z 23387527

[B29] Nieberler M. Reuning U. Reichart F. Notni J. Wester H.-J. Schwaiger M. (2017). Exploring the Role of RGD-Recognizing Integrins in Cancer. Cancers 9, 116. 10.3390/cancers9090116 PMC5615331 28869579

[B30] Pal K. Koner A. L. (2017). Rationally Designed Solvatochromic Fluorescent Indoline Derivatives for Probing Mitochondrial Environment. Chem. Eur. J. 23, 8610–8614. 10.1002/chem.201701425 28471005

[B31] Pierschbacher M. D. Ruoslahti E. (1984). Cell Attachment Activity of Fibronectin Can Be Duplicated by Small Synthetic Fragments of the Molecule. Nature 309, 30–33. 10.1038/309030a0 6325925

[B32] Raposo Moreira Dias A. Bodero L. Martins A. Arosio D. Gazzola S. Belvisi L. (2019). Synthesis and Biological Evaluation of RGD and isoDGR–Monomethyl Auristatin Conjugates Targeting Integrin αVβ3. Chem. Eur. J. 14, 938–942. 10.1002/cmdc.201900049 PMC6593765 30840356

[B33] Reynolds A. R. Hart I. R. Watson A. R. Welti J. C. Silva R. G. Robinson S. D. (2009). Stimulation of Tumor Growth and Angiogenesis by Low Concentrations of RGD-Mimetic Integrin Inhibitors. Nat. Med. 15, 392–400. 10.1038/nm.1941 19305413

[B34] Sancey L. Lucie S. Garanger E. Elisabeth G. Foillard S. Stéphanie F. (2009). Clustering and Internalization of Integrin Alphavbeta3 With a Tetrameric RGD-Synthetic Peptide. Mol. Ther. 17, 837–843. 10.1038/mt.2009.29 19259068 PMC2760123

[B35] Schneider H. Deweid L. Pirzer T. Yanakieva D. Englert S. Becker B. (2019a). Dextramabs: A Novel Format of Antibody‐Drug Conjugates Featuring a Multivalent Polysaccharide Scaffold. ChemistryOpen 8, 354–357. 10.1002/open.201900066 30976476 PMC6437811

[B36] Schneider H. Yanakieva D. Macarrón A. Deweid L. Becker B. Englert S. (2019b). TRAIL‐Inspired Multivalent Dextran Conjugates Efficiently Induce Apoptosis upon DR5 Receptor Clustering. ChemBioChem 20, 3006–3012. 10.1002/cbic.201900251 31206933

[B37] Takagi J. Petre B. M. Walz T. Springer T. A. (2002). Global Conformational Rearrangements in Integrin Extracellular Domains in Outside-In and Inside-Out Signaling. Cell 110, 599–611. 10.1016/S0092-8674(02)00935-2 12230977

[B38] Thumshirn G. Hersel U. Goodman S. L. Kessler H. (2003). Multimeric Cyclic RGD Peptides as Potential Tools for Tumor Targeting: Solid-Phase Peptide Synthesis and Chemoselective Oxime Ligation. Chem. Eur. J. 9, 2717–2725. 10.1002/chem.200204304 12772286

[B39] Tucci M. Stucci S. Silvestris F. (2014). Does Cilengitide Deserve Another Chance? Lancet Oncol. 15, e584–e585. 10.1016/S1470-2045(14)70462-0 25456376

[B40] Van Witteloostuijn S. B. Pedersen S. L. Jensen K. J. (2016). Half-Life Extension of Biopharmaceuticals Using Chemical Methods: Alternatives to PEGylation. ChemMedChem 11, 2474–2495. 10.1002/cmdc.201600374 27775236

[B41] Wu D. Zhao Z. Wang N. Zhang X. Yan H. Chen X. (2020). Fluorescence Imaging-Guided Multifunctional Liposomes for Tumor-specific Phototherapy for Laryngeal Carcinoma. Biomater. Sci. 8, 3443–3453. 10.1039/D0BM00249F 32412569

[B42] Yousefi H. Vatanmakanian M. Mahdiannasser M. Mashouri L. Alahari N. V. Monjezi M. R. (2021). Understanding the Role of Integrins in Breast Cancer Invasion, Metastasis, Angiogenesis, and Drug Resistance. Oncogene 40, 1043–1063. 10.1038/s41388-020-01588-2 33420366

[B43] Zarovni N. Monaco L. Corti A. (2004). Inhibition of Tumor Growth by Intramuscular Injection of cDNA Encoding Tumor Necrosis FactorαCoupled to NGR and RGD Tumor-Homing Peptides. Hum. Gene Ther. 15, 373–382. 10.1089/104303404322959524 15053862

