# Supplementary material for: Synthetic Integrin-Targeting Dextran-Fc Hybrids Efficiently Inhibit Tumor Proliferation In Vitro
Source: Front Chem. 2021 Jul 22;9:693097. doi: 10.3389/fchem.2021.693097 (PMC8339797; doi:10.3389/fchem.2021.693097)
Supplement: Supplementary file 1 [file DataSheet1.PDF]

Supplementary Material

1 Overview of synthesized compounds 2
2 Synthesis and Analytical Data 4
2.1 Synthesis of 4-Pentynoic Acid NHS Ester **17** 4
2.2 Synthetic Approach to C*yclo*[RGDfK(4-Pentynoic Acid)] **18** and *Cyclo*[RADfK(4-Pentynoic Acid)] **19** 6
2.2.1 Analytical Data of *H*-D(OtBu)fK(Boc)R(Pbf)G-*OH* **11** 6
2.2.2 Analytical Data of *H*-D(OtBu)fK(Boc)R(Pbf)A-*OH* **12** 8
2.2.3 Analytical Data of *Cyclo*[D(OtBu)fK(Boc)R(Pbf)G] **13** 9
2.2.4 Analytical Data of *Cyclo*[D(OtBu)fK(Boc)R(Pbf)A] **14** 11
2.2.5 Analytical Data of *Cyclo*[RGDfK] **15** 13
2.2.6 Analytical Data of *Cyclo*[RADfK] **16** 15
2.2.7 Analytical Data of *Cyclo*[RGDfK(4-pentynoic acid)] **18** 18
2.2.8 Analytical Data of *Cyclo*[RADfK(4-pentynoic acid)] **19** 20
2.3 Synthetic Approach to Cadaverine-Dextran-$(N_3)_{15.6}$ **9** 22
2.4 Sortase A-Mediated Conjugation of MMAE to Fc **1** 23
2.5 Transglutaminase-Catalyzed Conjugation of Dextran 27
2.6 CuAAC-Catalyzed Cycloaddition 29
3 Thermal Shift Assay 31
4 K_D on U87MG cells 32
5 Literature 33

1 Overview of synthesized compounds

Supplementary Table 1: Overview of generated compounds.

Number	Compound	Description
**1**	Fc(N297A)	Aglycosylated Fc fragment
**3**	Fc-MMAE	MMAE conjugated to Fc via Sortase A
**5**	*N*-Boc-cadaverine-dextran	10 kDa Dextran modified with *N*-Boc-cadaverine at its reducing end
**6**	*N*-Boc-cadaverine-dextran-$CED_{15.6}$	*N*-Boc-cadaverine-dextran with 15.6 carboxyethyl groups at the C-2 hydroxy groups
**7**	*N*-(5-Aminopentyl)-2-azido-acetamide	Azide linker
**8**	*N*-Boc-cadaverine-dextran-$(N_3)_{15.6}$	*N*-Boc-cadaverine-dextran with 15.6 azide linker at the C-2 hydroxy groups
**9**	Cadaverine-dextran-$(N_3)_{15.6}$	Cadaverine-dextran with 15.6 azide linker at the C-2 hydroxy groups
**10**	Fc-MMAE-dextran	Fc-MMAE **3** functionalized with dextran **9**
**11**<br>**12**	*H*-D(OtBu)fK(Boc)R(Pbf)G-*OH*<br>*H*-D(OtBu)fK(Boc)R(Pbf)A-*OH*	Linear pentapeptides with side-chain protecting groups
**13**<br>**14**	*Cyclo*[D(OtBu)fK(Boc)R(Pbf)G]<br>*Cyclo*[D(OtBu)fK(Boc)R(Pbf)A]	Cyclized pentapeptides with side-chain protecting groups
**15**<br>**16**	*Cyclo*[RGDfK]<br>*Cyclo*[RADfK]	Cyclized pentapeptides
**17**	4-pentynoic acid NHS ester	-

**18**<br>**19**	*Cyclo*[RGDfK(4-pentynoic acid)]<br>*Cyclo*[RADfK(4-pentynoic acid)]	Alkyne-bearingcCyclized pentapeptide
**20**	Fc-MMAE-dextran-RGD	RGD-decorated Fc-MMAE-dextran
**21**	Fc-MMAE-dextran-RAD	RAD-decorated Fc-MMAE-dextran
**22**	Fc-dextran	Fc-fragment **1** functionalized with dextran **9**
**23**	Fc-dextran-RGD	RGD-decorated Fc-dextran
**24**	Fc-dextran-RAD	RAD-decorated Fc-dextran

2 Synthesis and Analytical Data

2.1 Synthesis of 4-Pentynoic Acid NHS Ester 17

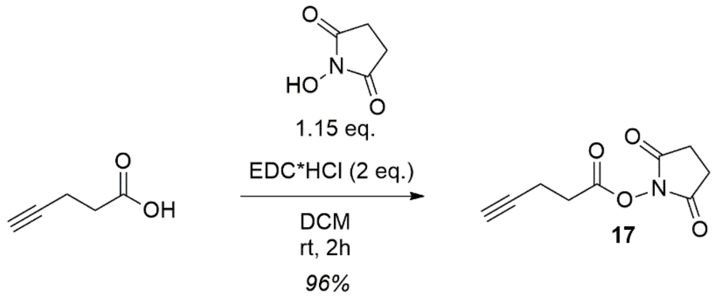

Supplementary Figure 1: Synthesis of 4-pentynoic acid NHS ester **17.**

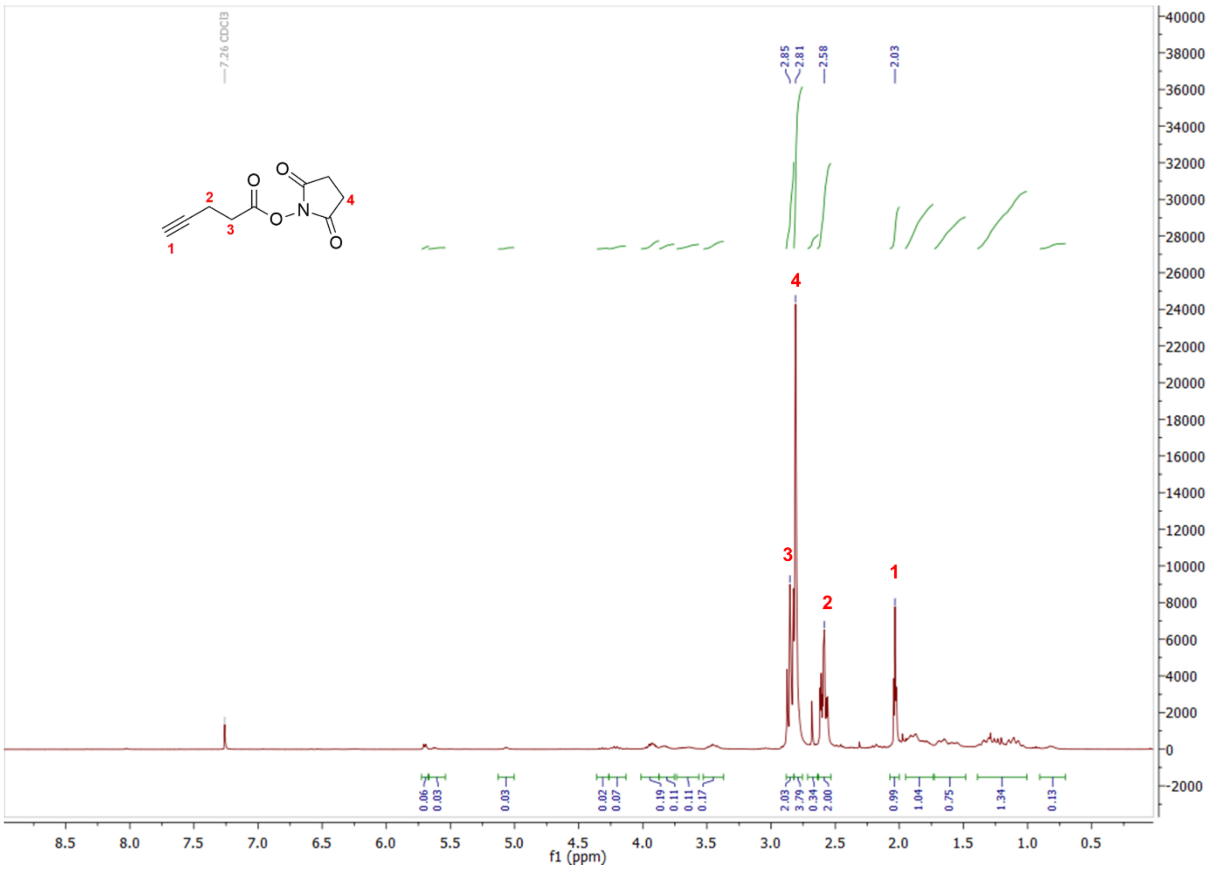

Supplementary Figure 2: ^{1}H-NMR (300 MHz, $CDCl_3$) of 4-pentynoic acid NHS ester **17**.

^{1}H NMR (300 MHz, $CDCl_3$) δ = 2.90-2.82 (m, 2H), 2.81 (s, 4H), 2.64-2.54 (m, 2H), 2.03 (t, J = 2.6 Hz, 1H) ppm.

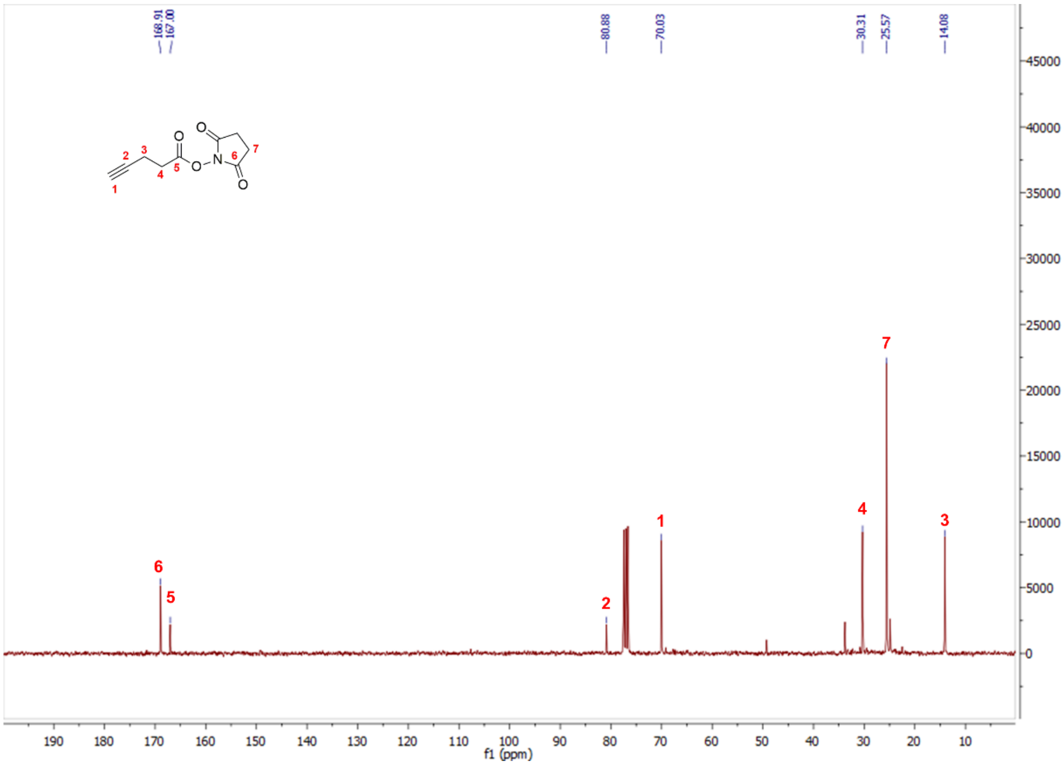

Supplementary Figure 3: ^{13}C-NMR (75 MHz, $CDCl_3$) of 4-pentynoic acid-NHS **17**.

^{13}C NMR (75 MHz, $CDCl_3$) δ = 171.7, 168.9, 82.2, 70.9, 31.2, 26.4, 14.7 ppm.

2.2 Synthetic Approach to C*yclo*[RGDfK(4-Pentynoic Acid)] 18 and *Cyclo*[RADfK(4-Pentynoic Acid)] 19

2.2.1 Analytical Data of *H*-D(OtBu)fK(Boc)R(Pbf)G-*OH* 11

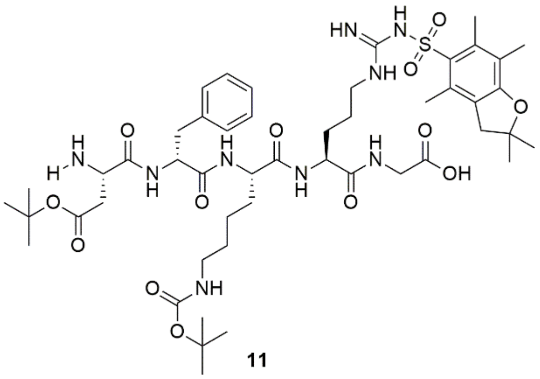

11

$C_{49}H_{75}N_9O_{13}S$

M_w = 1030.24 gmol^{-1}

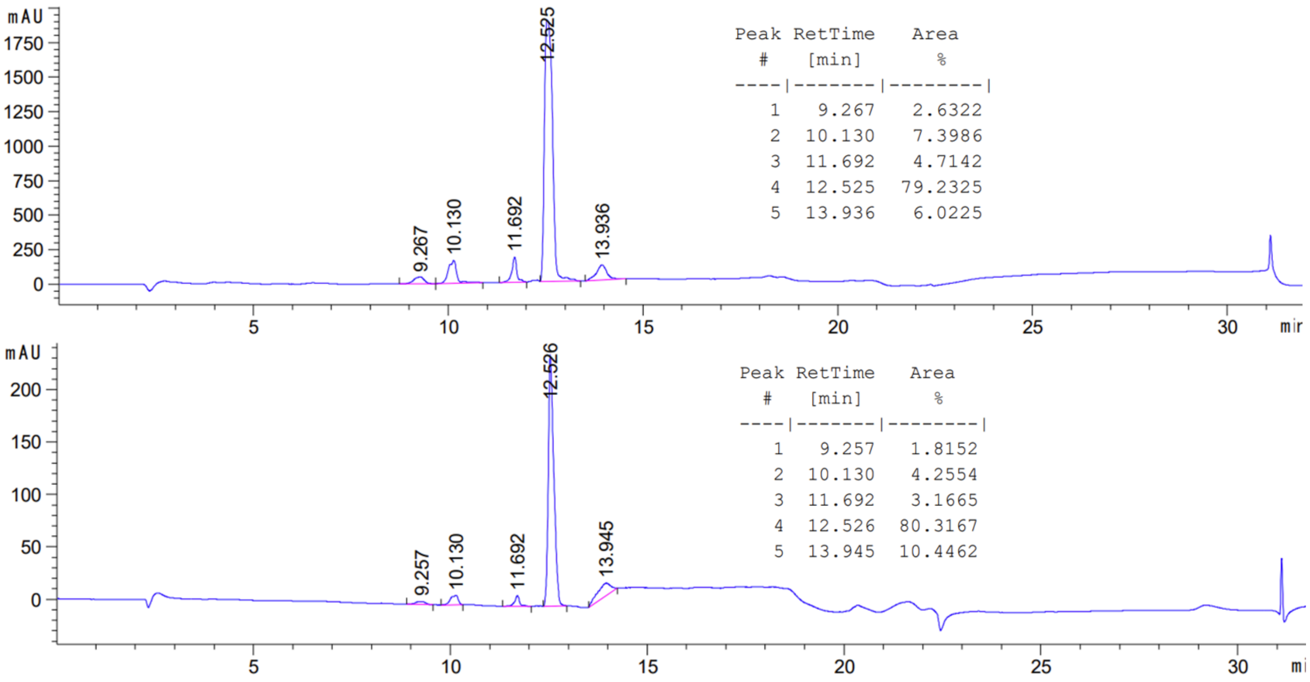

Supplementary Figure 4: Chromatographic trace of *H*-D(OtBu)fK(Boc)R(Pbf)G-*OH* **11,** 30 to 100 % B (gradient 20 min), λ = 220 nm (top), λ = 280 nm (bottom), t_R = 12.525 min.

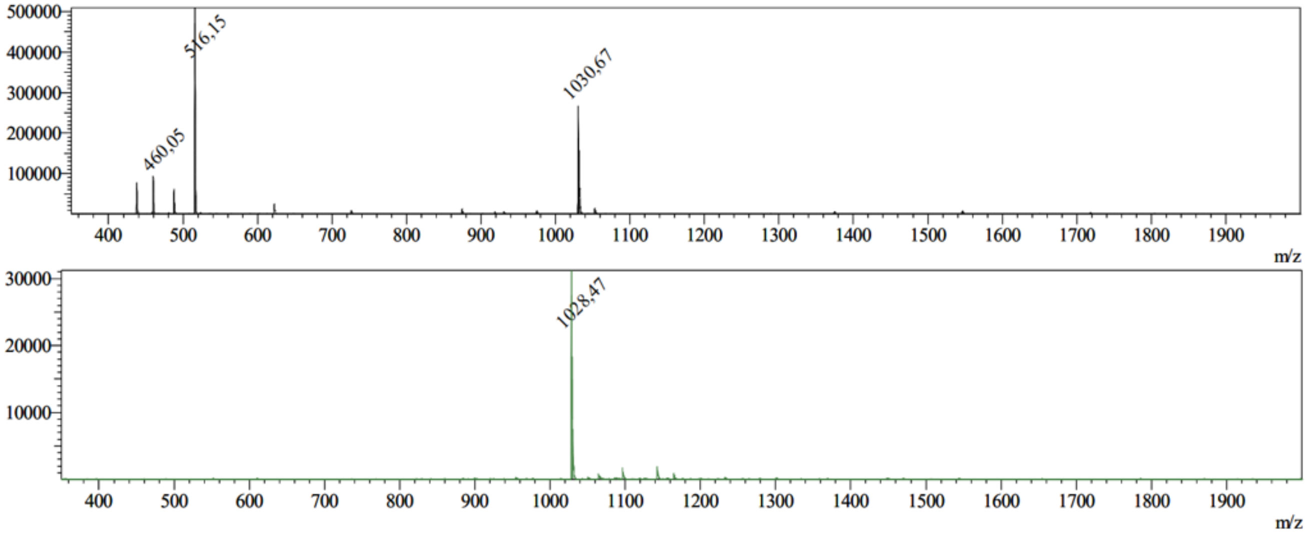

Supplementary Figure 5: ESI-MS of *H*-D(OtBu)fK(Boc)R(Pbf)G-*OH* **11**; m/$z_{calc.}$ = 1030.53 $[M+H]^+$ m/$z_{obs.}$ = 1030.67, m/$z_{calc.}$ = 1028.51 $[M-H]^-$ m/$z_{obs.}$ = 1028.47.

2.2.2 Analytical Data of *H*-D(OtBu)fK(Boc)R(Pbf)A-*OH* 12

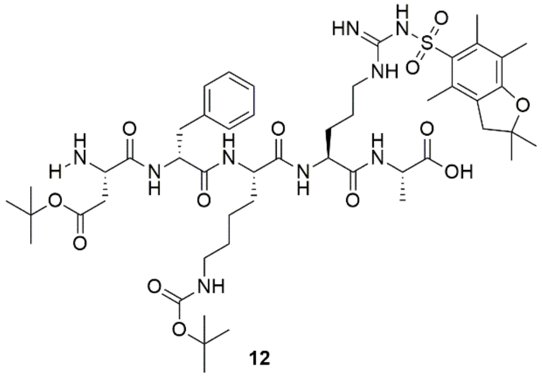

$C_{50}H_{77}N_{9}O_{13}S$

Mw = 1044.26 $gmol^{-1}$

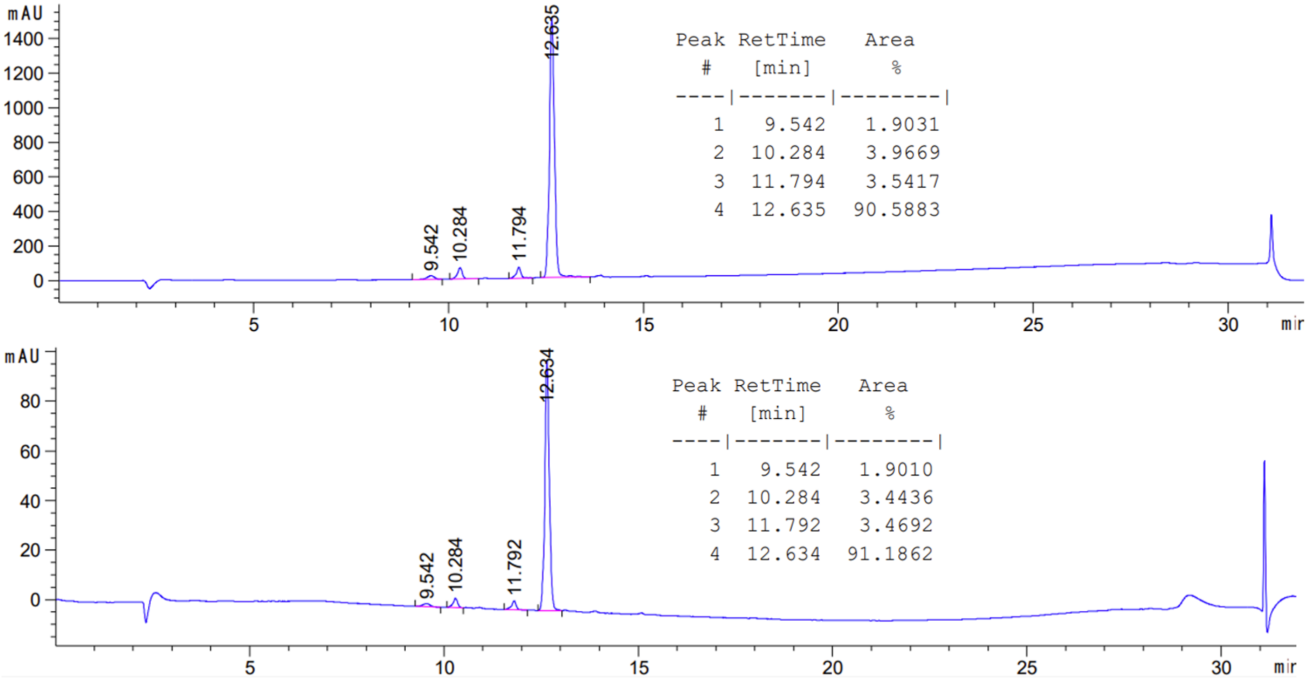

Supplementary Figure 6: Chromatographic trace of *H*-D(OtBu)fK(Boc)R(Pbf)A-*OH* **12,** 30 to 100 % B (gradient 20 min), λ = 220 nm (top), λ = 280 nm (bottom), t_R = 12.635 min.

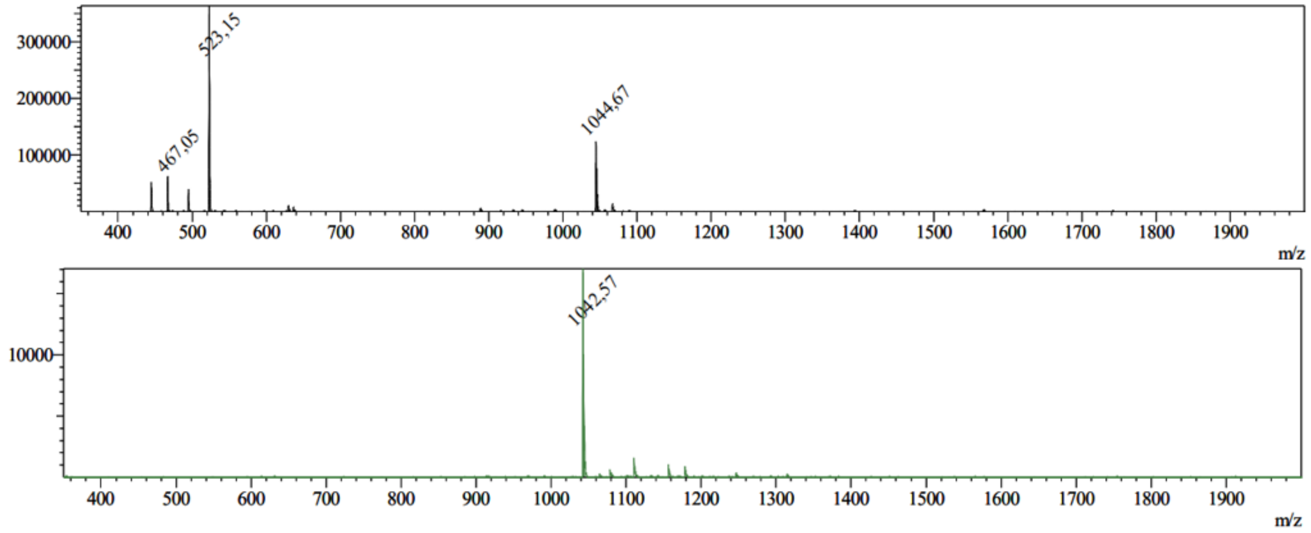

Supplementary Figure 7: ESI-MS of *H*-D(OtBu)fK(Boc)R(Pbf)G-*OH* **12**; m/$z_{calc.}$ = 1044.54 $[M+H]^+$ m/$z_{obs.}$ = 1044.67, m/$z_{calc.}$ = 1042.53 $[M-H]^-$ m/$z_{obs.}$ = 1042.57.

2.2.3 Analytical Data of *Cyclo*[D(OtBu)fK(Boc)R(Pbf)G] 13

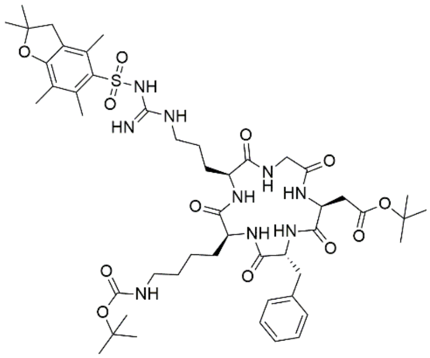

13

$C_{49}H_{73}N_9O_{12}S$

M_w = 1012.22 $gmol^{-1}$

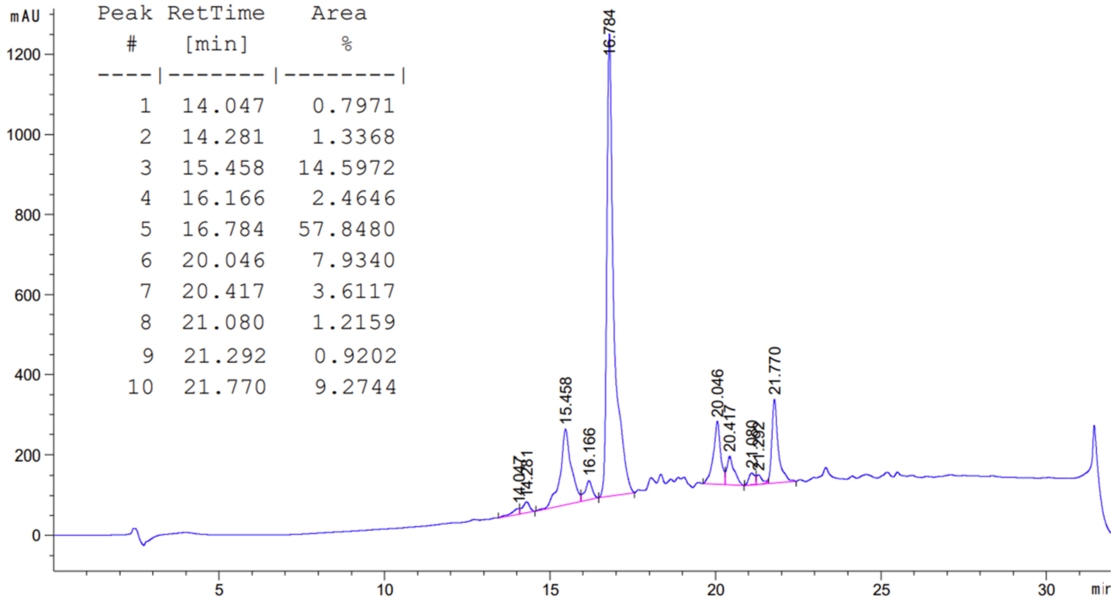

Supplementary Figure 8: Chromatographic trace of *cyclo*[D(OtBu)fK(Boc)R(Pbf)G] **13**, 30 to 100 % B (gradient 20 min), λ = 220 nm, t_R = 16.784 min.

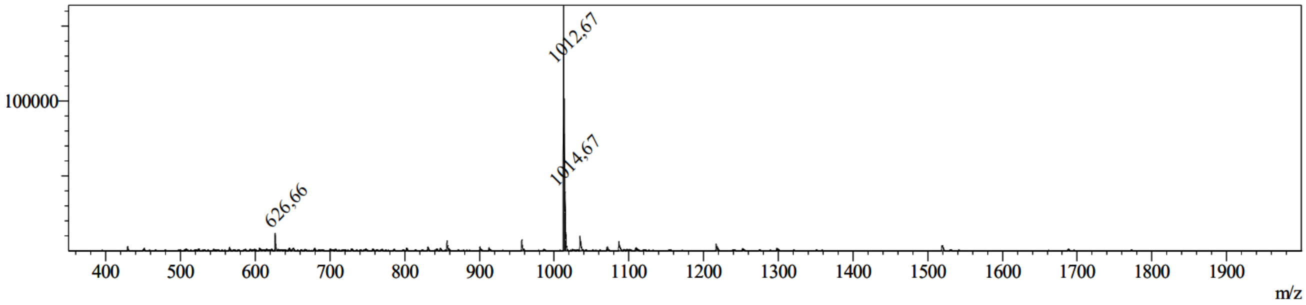

Supplementary Figure 9: ESI-MS of *cyclo*[D(OtBu)fK(Boc)R(Pbf)G] **13**; $m/z_{calc.}$ = 1012.52 $[M+H]^+$ $m/z_{obs.}$ = 1012.67.

2.2.4 Analytical Data of *Cyclo*[D(OtBu)fK(Boc)R(Pbf)A] 14

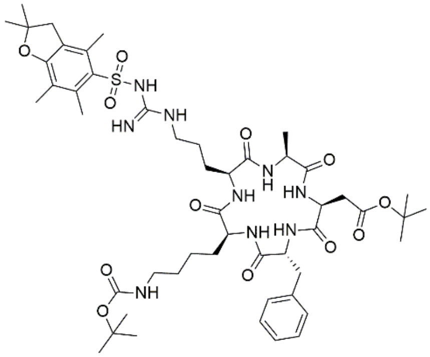

14

$C_{50}H_{75}N_9O_{12}S$

M_w = 1026.25 gmol^{-1}

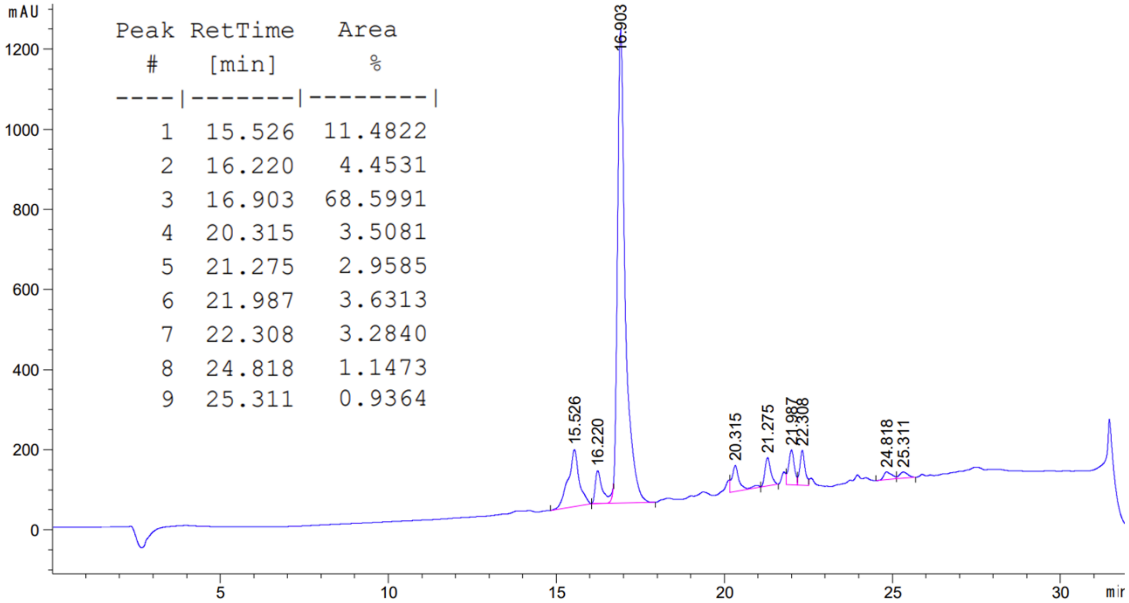

Supplementary Figure 10: Chromatographic trace of *cyclo*[D(OtBu)fK(Boc)R(Pbf)A] **14**, 30 to 100 % B (gradient 20 min), λ = 220 nm, t_R = 16.903 min.

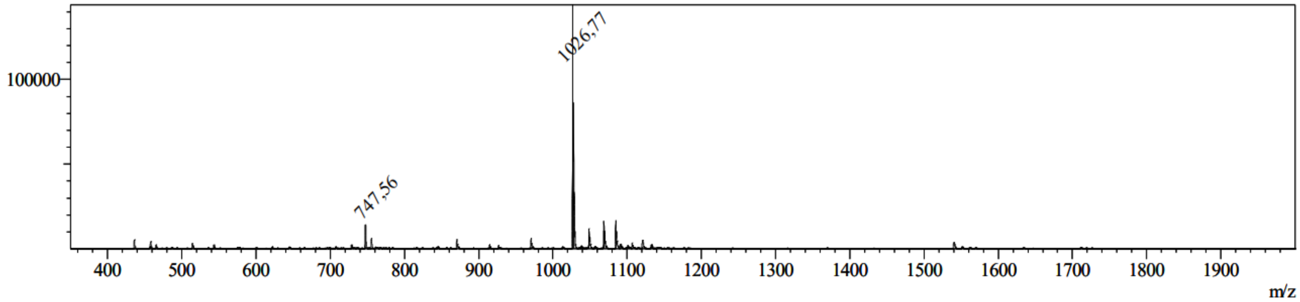

Supplementary Figure 11: ESI-MS of *cyclo*[D(OtBu)fK(Boc)R(Pbf)A] **14**; m/$z_{calc.}$ = 1026.53 $[M+H]^+$ m/$z_{obs.}$ = 1026.77.

2.2.5 Analytical Data of *Cyclo*[RGDfK] 15

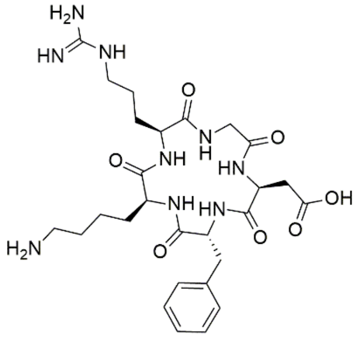

15

$C_{27}H_{41}N_9O_7$

M_w = 603.67 $gmol^{-1}$

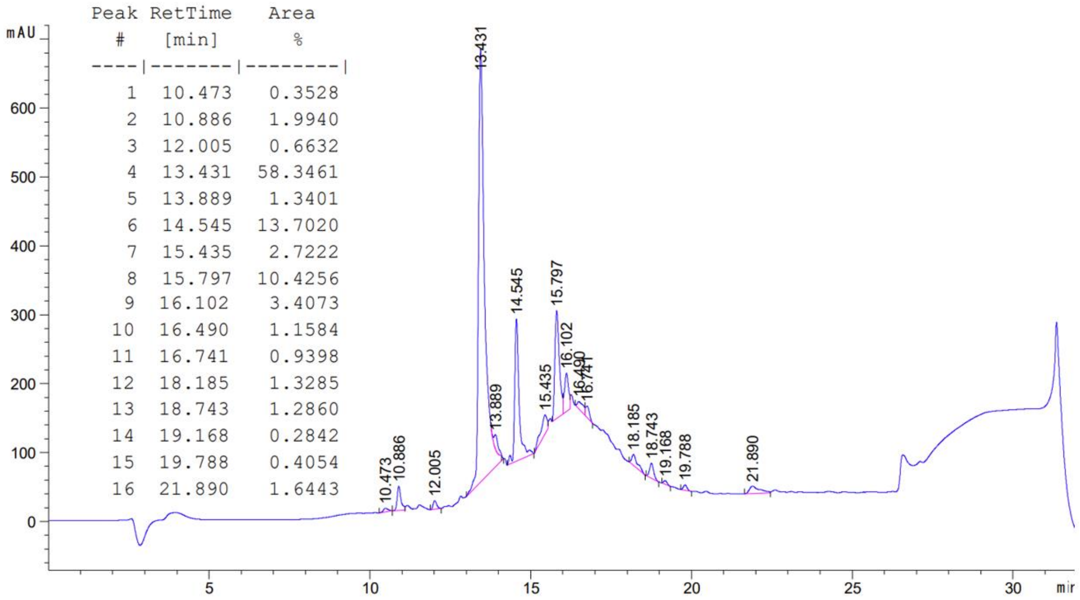

Peak #	RetTime [min]	Area %
1	10.473	0.3528
2	10.886	1.9940
3	12.005	0.6632
4	13.431	58.3461
5	13.889	1.3401
6	14.545	13.7020
7	15.435	2.7222
8	15.797	10.4256
9	16.102	3.4073
10	16.490	1.1584
11	16.741	0.9398
12	18.185	1.3285
13	18.743	1.2860
14	19.168	0.2842
15	19.788	0.4054
16	21.890	1.6443

Supplementary Figure 12: Chromatographic trace of crude *cyclo*[RGDfK] **15**, 0 to 40 % B (gradient 20 min), λ = 220 nm, t_R = 13.431 min.

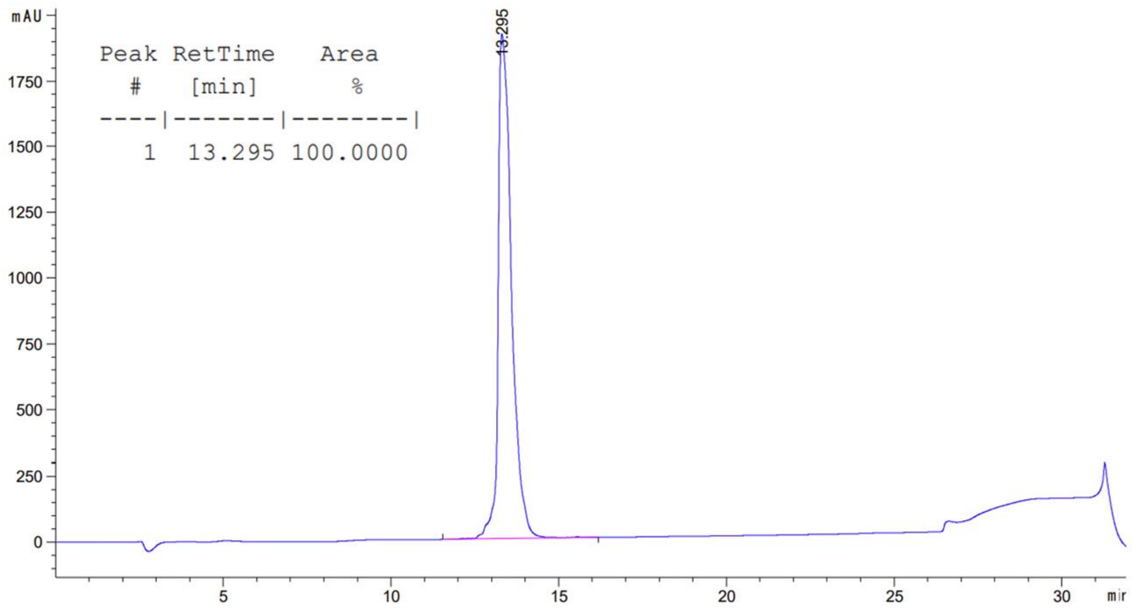

Supplementary Figure 13: Chromatographic trace of *cyclo*[RGDfK] **15**, 0 to 40 % B (gradient 20 min), λ = 220 nm, t_R = 13.295 min.

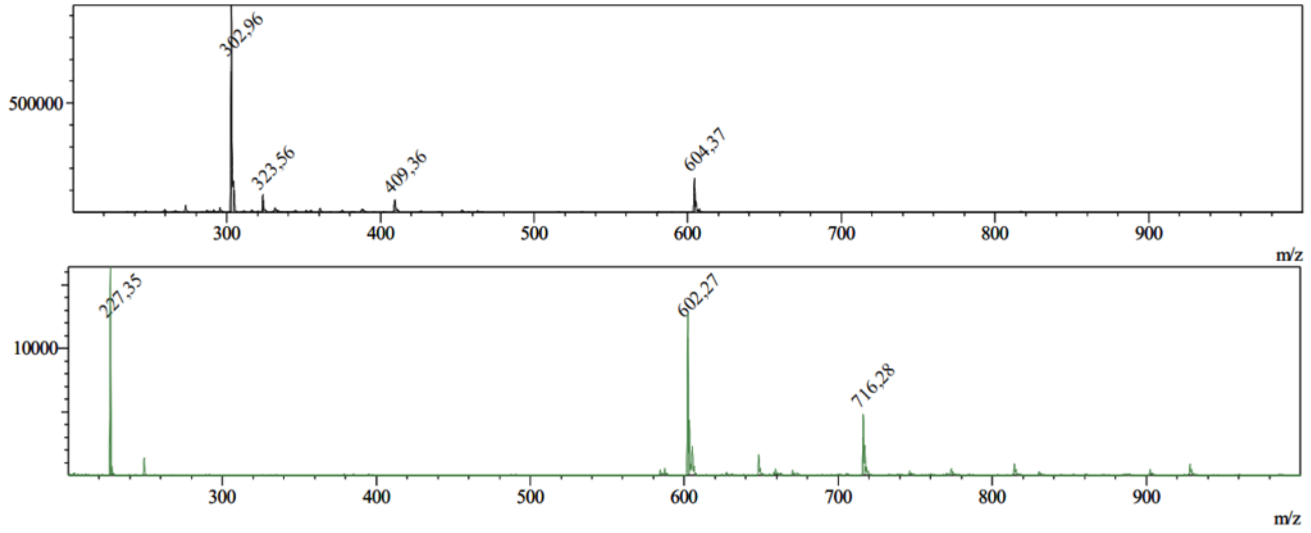

Supplementary Figure 14: ESI-MS of *cyclo*[RGDfK] **15**; $m/z_{calc.}$ = 604.32 $[M+H]^+$ $m/z_{obs.}$ = 604.37, $m/z_{calc.}$ = 602.31 $[M-H]^-$ $m/z_{obs.}$ = 602.27.

2.2.6 Analytical Data of *Cyclo*[RADfK] 16

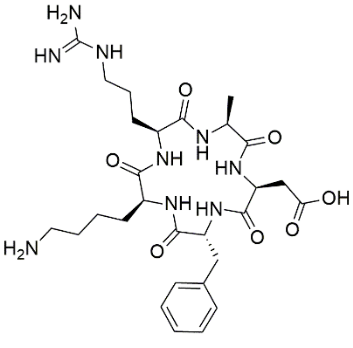

16

$C_{28}H_{43}N_9O_7$

M_w = 617.70 $gmol^{-1}$

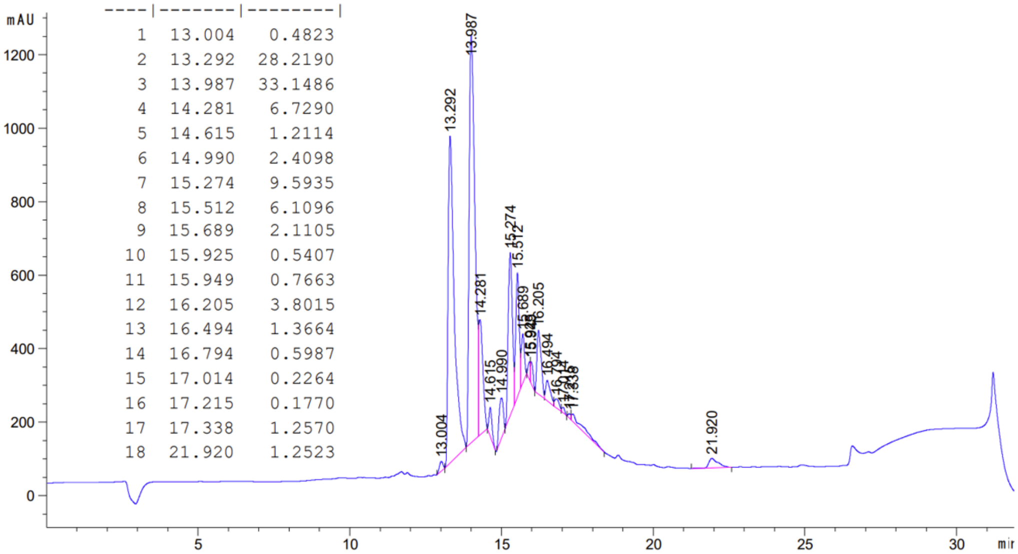

Peak #	RetTime [min]	Area %
1	13.004	0.4823
2	13.292	28.2190
3	13.987	33.1486
4	14.281	6.7290
5	14.615	1.2114
6	14.990	2.4098
7	15.274	9.5935
8	15.512	6.1096
9	15.689	2.1105
10	15.925	0.5407
11	15.949	0.7663
12	16.205	3.8015
13	16.494	1.3664
14	16.794	0.5987
15	17.014	0.2264
16	17.215	0.1770
17	17.338	1.2570
18	21.920	1.2523

Supplementary Figure 15: Chromatographic trace of crude *cyclo*[RADfK] **16** containing two isomers (t_R = 13.292 min, 13.987 min, ratio 46:54 according to area under the curve), 0 to 40 % B (gradient 20 min), λ = 220 nm.

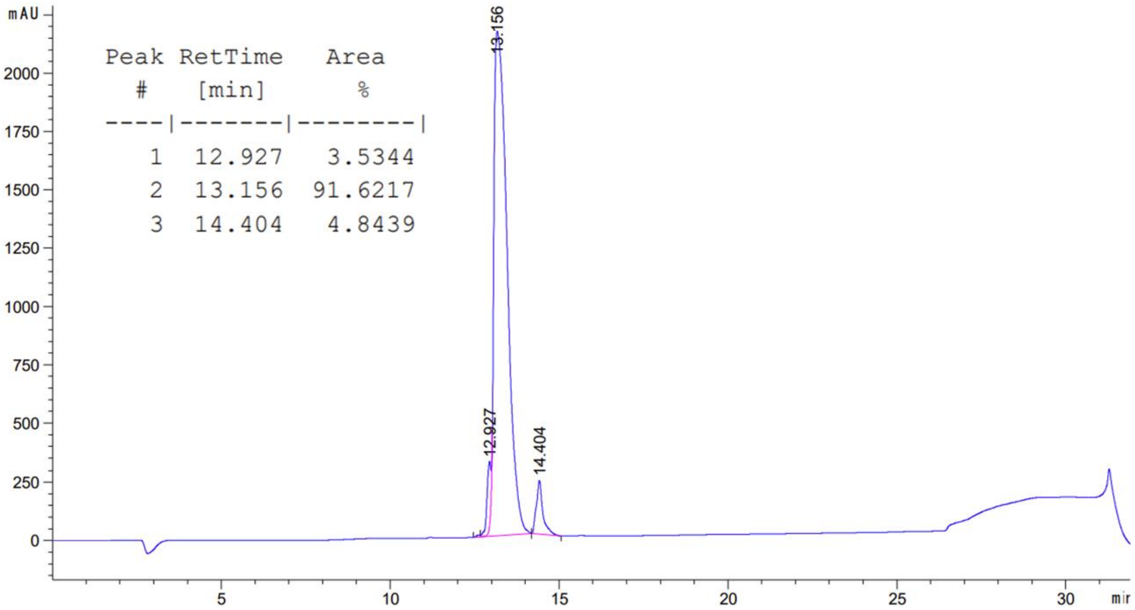

Supplementary Figure 16: Chromatographic trace of first isomer of *cyclo*[RADfK] **16** (t_R = 13.156 min) with traces of second isomer (t_R = 14.404 min), 0 to 40 % B (gradient 20 min), λ = 220 nm.

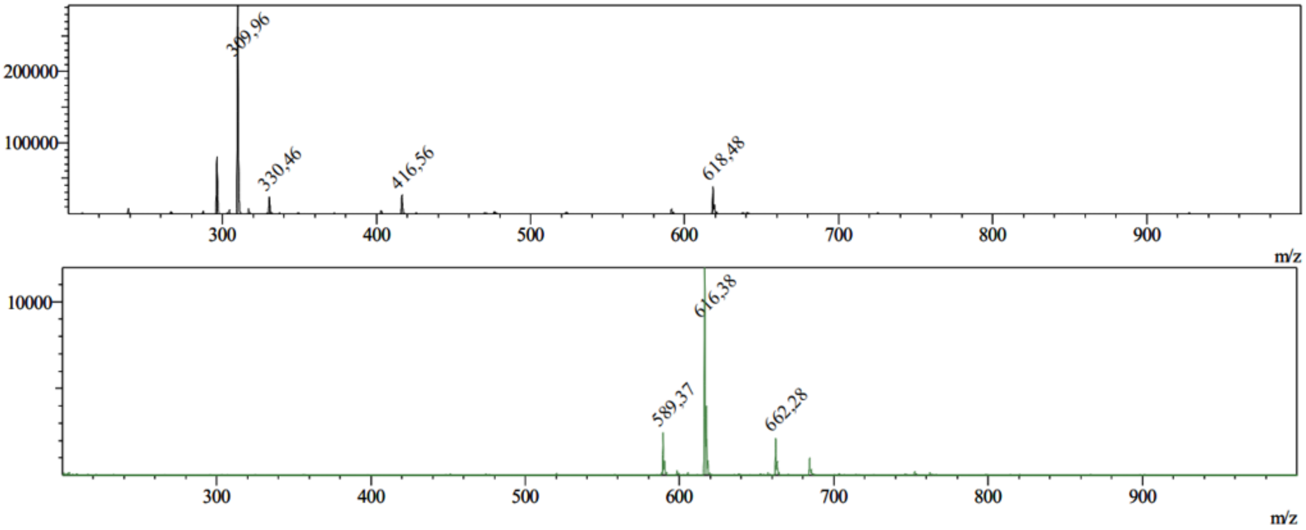

Supplementary Figure 17: ESI-MS of first isomer (corresponds to lower retention time) of *cyclo*[RADfK] **16**; $m/z_{calc.}$ = 618.34 $[M+H]^+$ $m/z_{obs.}$ = 618.48, $m/z_{calc.}$ = 616.32 $[M-H]^-$ $m/z_{obs.}$ = 616.38.

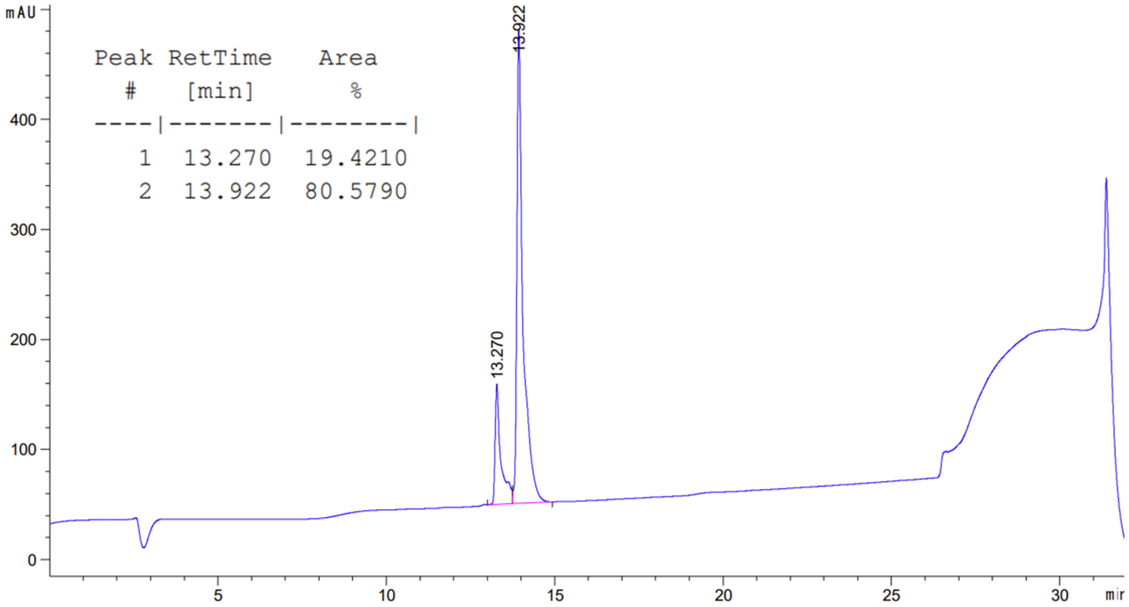

Supplementary Figure 18: Chromatographic trace of second isomer of *cyclo*[RADfK] **16** (t_R = 13.922 min.) with traces of first isomer (t_R = 13.270 min.), 0 to 40 % B (gradient 20 min), λ = 220 nm,

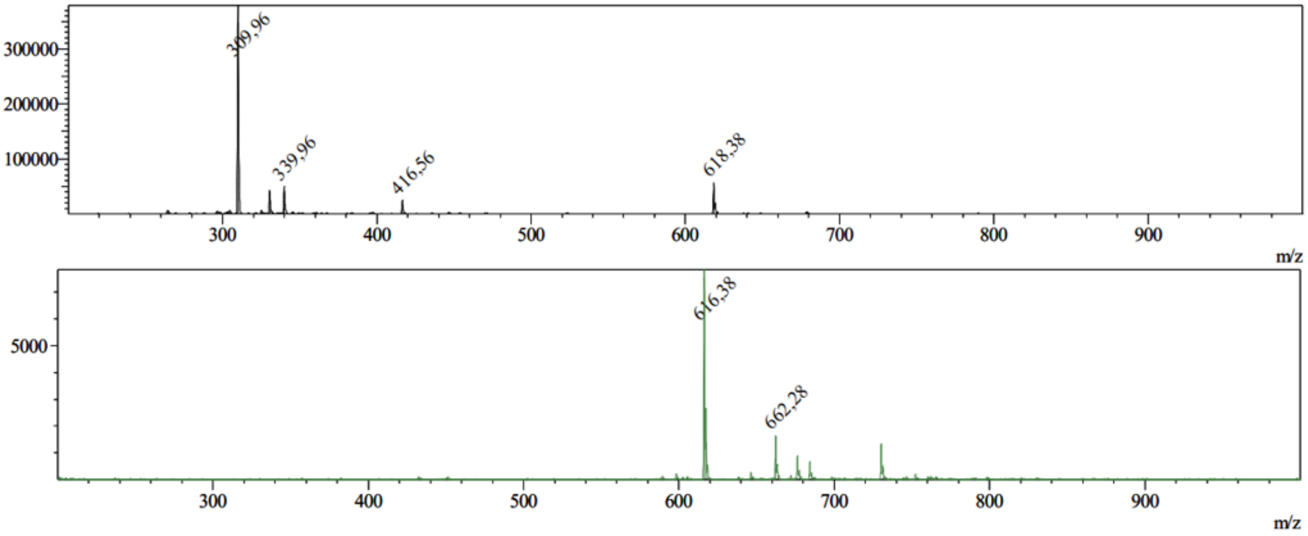

Supplementary Figure 19: ESI-MS of second isomer (corresponds to higher retention time) of *cyclo*[RADfK] **16**; $m/z_{calc.}$ = 618.34 $[M+H]^+$ $m/z_{obs.}$ = 618.38, $m/z_{calc.}$ = 616.32 $[M-H]^-$ $m/z_{obs.}$ = 616.38.

2.2.7 Analytical Data of *Cyclo*[RGDfK(4-pentynoic acid)] 18

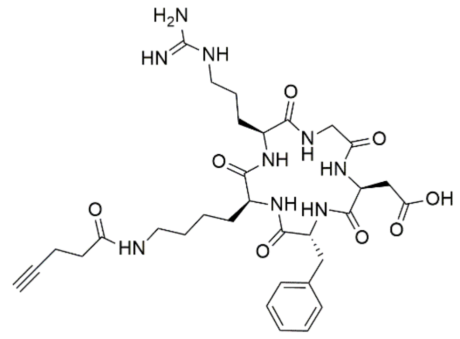

18

$C_{32}H_{45}N_9O_8$

M_w = 683.84 gmol^{-1}

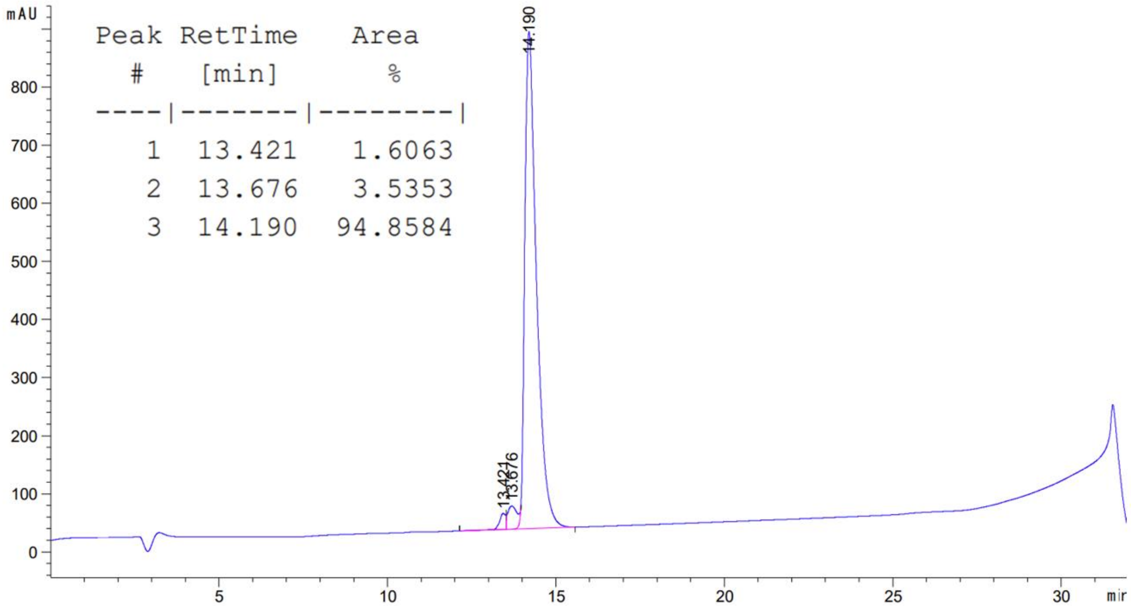

Supplementary Figure 20: Chromatographic trace of *cyclo*[RGDfK(4-pentynoic acid)] **18**, 10 to 50 % B (gradient 20 min), λ = 220 nm, t_R = 14.190 min.

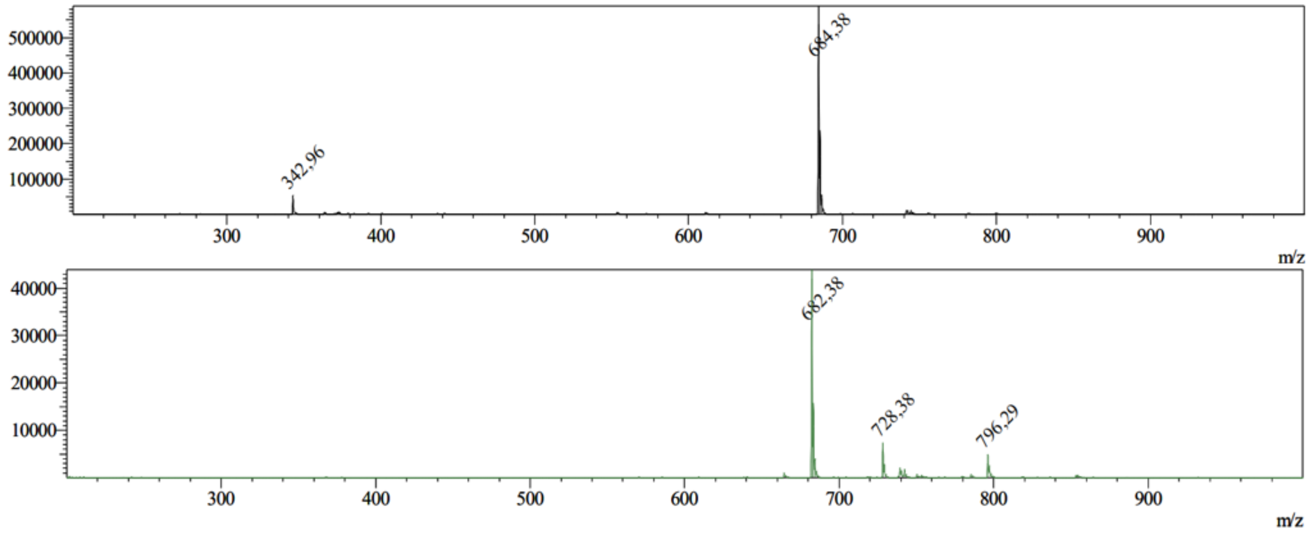

Supplementary Figure 21: ESI-MS of *cyclo*[RGDfK(4-pentynoic acid)] **18**; $m/z_{calc.}$ = 684.35 $[M+H]^+$ $m/z_{obs.}$ = 684.38, $m/z_{calc.}$ = 682.33 $[M-H]^-$ $m/z_{obs.}$ = 682.38.

2.2.8 Analytical Data of *Cyclo*[RADfK(4-pentynoic acid)] 19

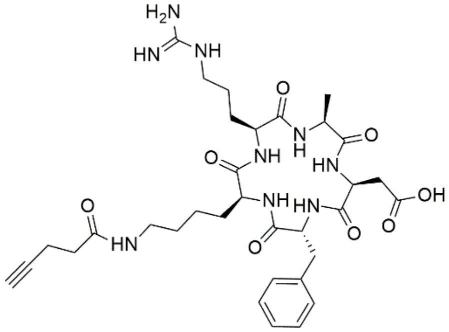

19

$C_{33}H_{47}N_9O_8$

M_w = 697.78 gmol^{-1}

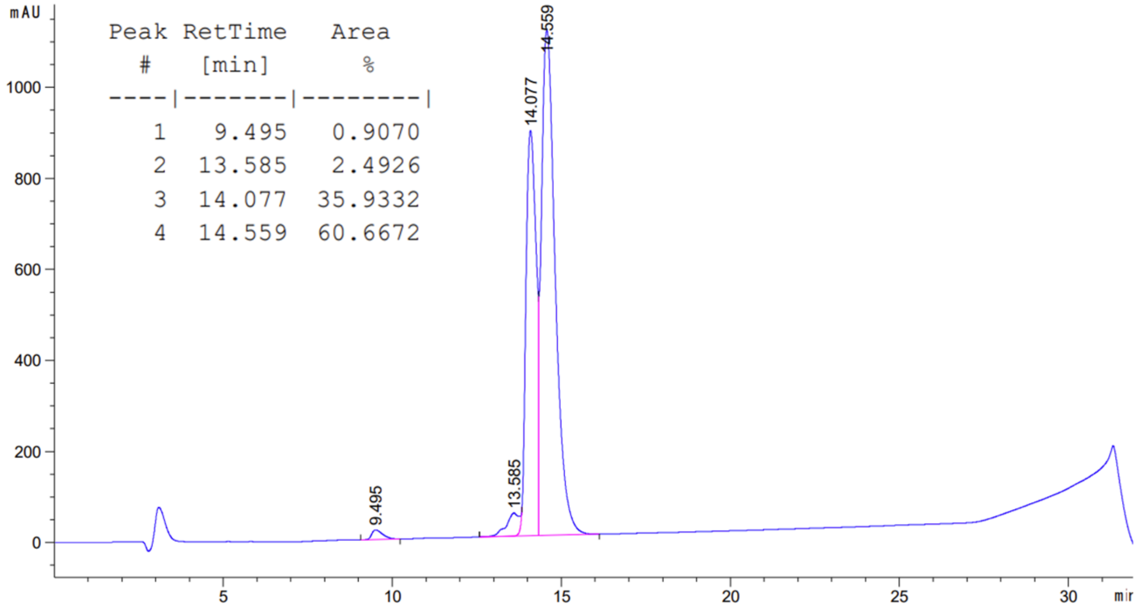

Supplementary Figure 22: Chromatographic trace of *cyclo*[RADfK(4-pentynoic acid)] **19**, 10 to 50 % B (gradient 20 min), λ = 220 nm, t_R = 14.077 min, 14.559 min (corresponding to both isomers of educt c[RADfK] **17**).

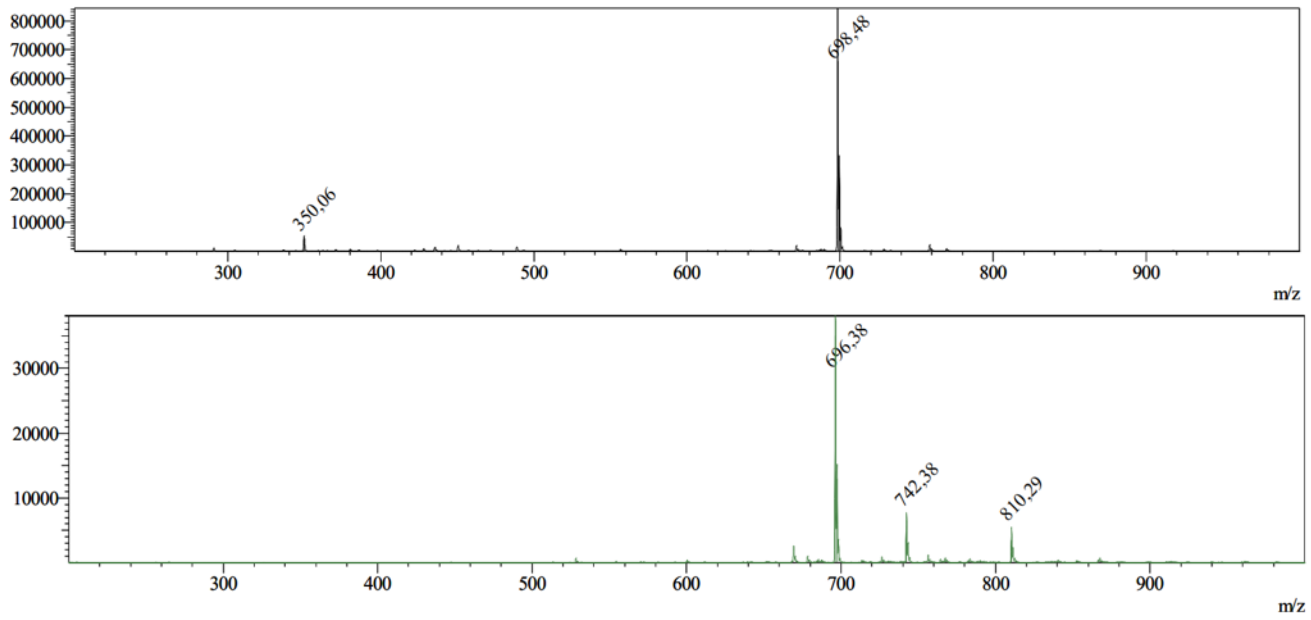

Supplementary Figure 23: ESI-MS of *cyclo*[RADfK(4-pentynoic acid)] **19**; $m/z_{calc.}$ = 698.36 $[M+H]^+$ $m/z_{obs.}$ = 698.48, $m/z_{calc.}$ = 696.35 $[M-H]^-$ $m/z_{obs.}$ = 696.35.

2.3 Analytical Data of Cadaverine-Dextran-$(N_3)_{15.6}$ 9

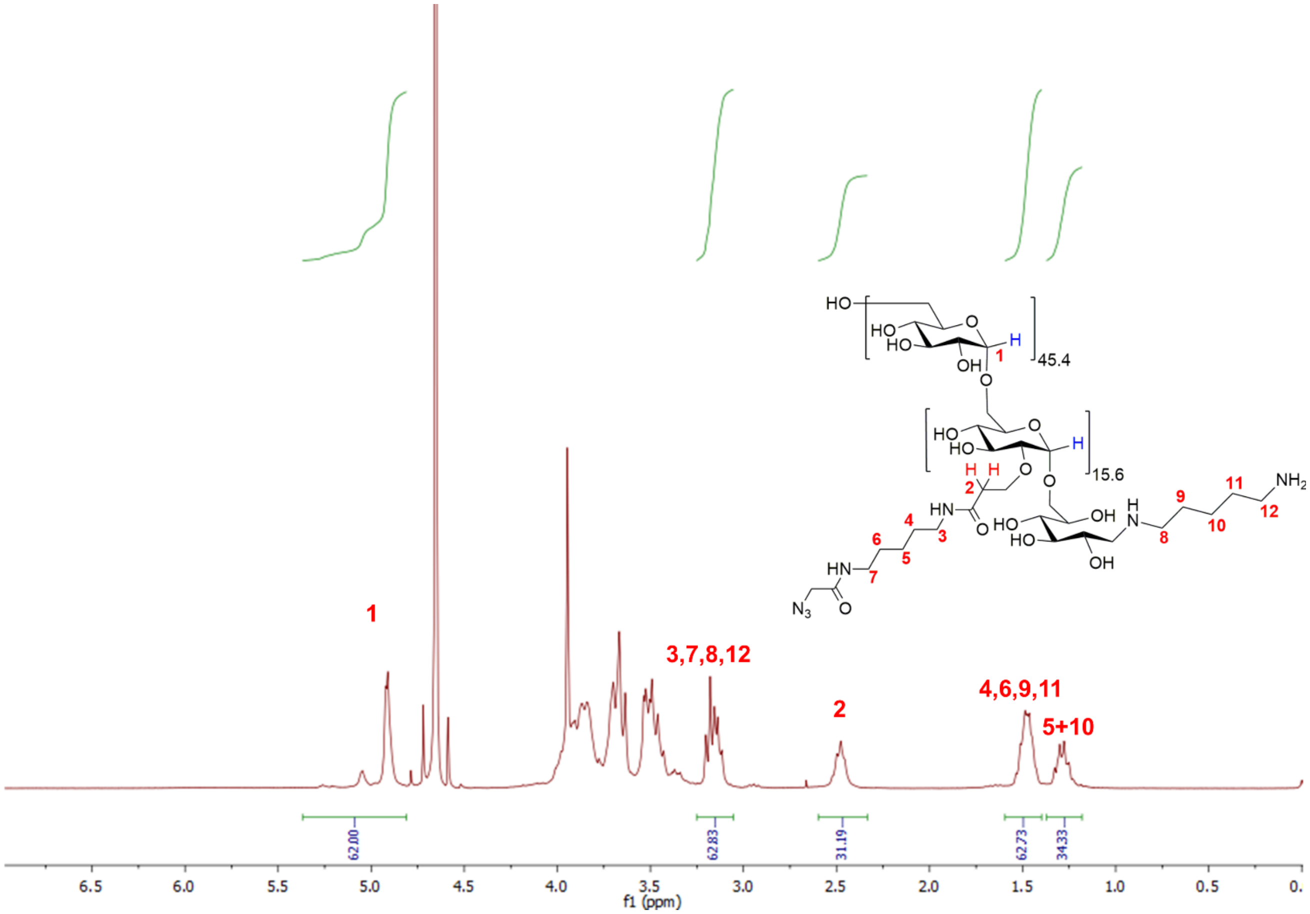

Supplementary Figure 24: ^{1}H-NMR (300 MHz, D_2O) of cadaverine-dextran$(N_3)_{15.6}$ **9** with corresponding structure. Anomeric protons are displayed in blue, unobstructed protons of the carboxyethyl function are shown in red.

For quantification of carboxyethyl groups, the integral of anomeric protons is set to 62 corresponding to the average of number of glucose units in 10 kDa dextran. The number of carboxyethyl groups results from the integral of H-2 divided by two (corresponding to two protons). For further analysis IR-spectroscopy was performed to ensure qualitatively the presence of azides in the product.

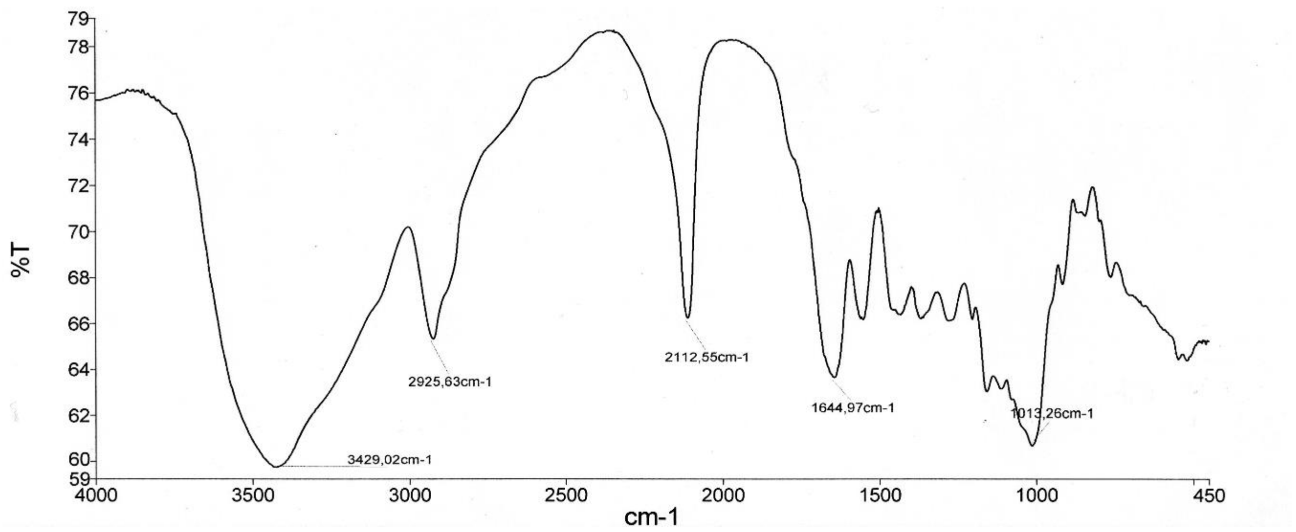

Supplementary Figure 25: IR-spectrum of cadaverine-dextran$(N_3)_{15.6}$ **9** (measured as KBr pellet). The azide band is at a wavenumber of 2112.55 cm^{-1}.

2.4 Sortase A-Mediated Conjugation of MMAE to Fc 1

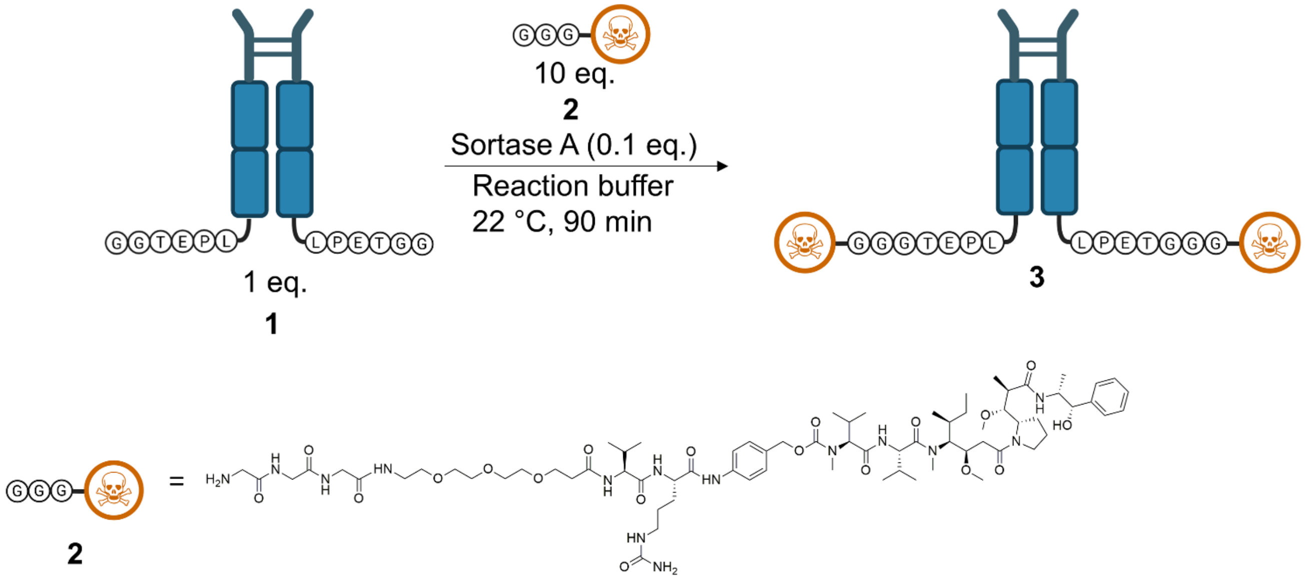

Supplementary Figure 26: Sortase A-mediated conjugation of MMAE to Fc **1** leading to Fc-MMAE **3**. (Reaction buffer: Tris 50 mM, NaCl 150 mM, $CaCl_2$ 10 mM, pH 7.5). Scheme was created with biorender.com.

For reaction control, HIC analysis was performed. As can be seen in the following figure (Supplementary Figure 27), conjugation of MMAE leads to two peaks with higher retention time, which corresponds to Fc with a single MMAE coupled and Fc with two MMAE coupled (**3**), respectively.

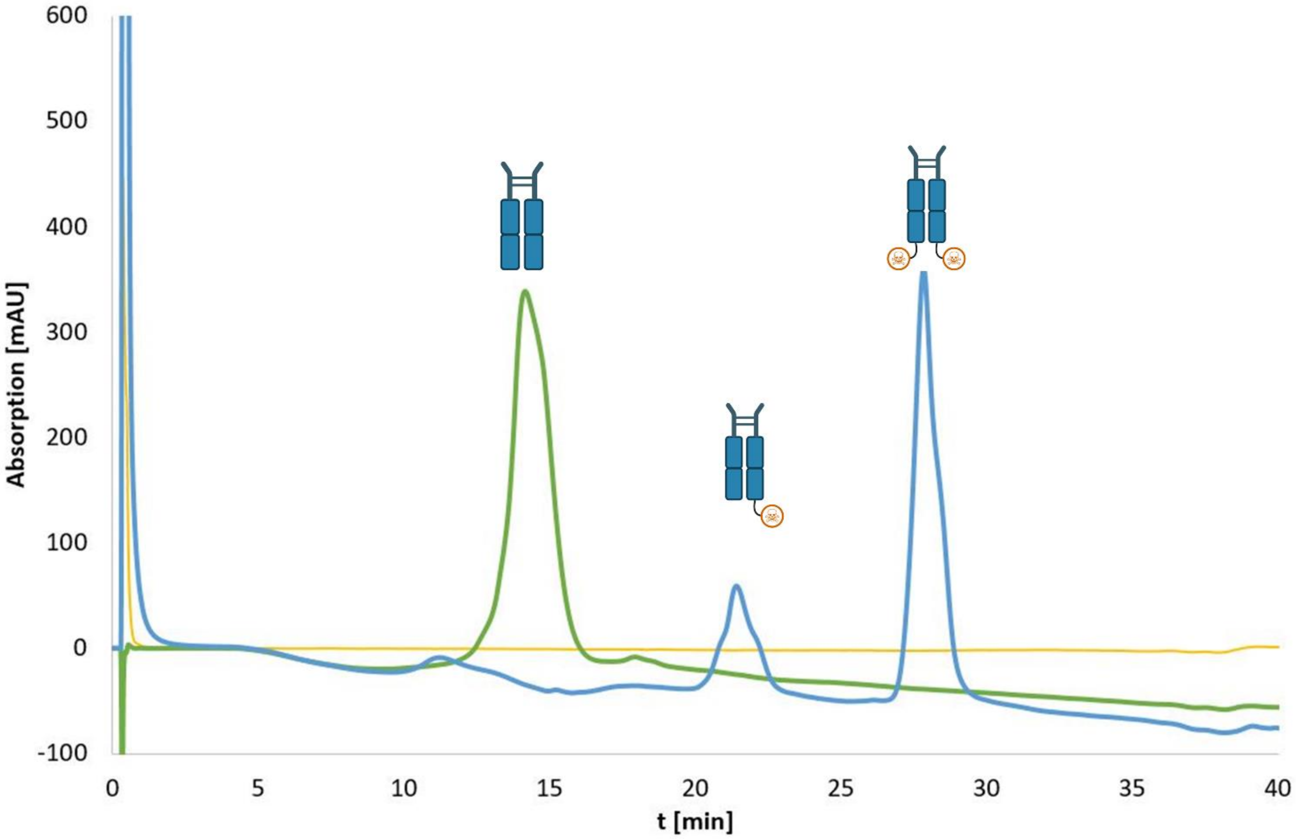

Supplementary Figure 27: HIC traces of sortase A (yellow), Fc **1** (green) and Fc-MMAE **3** (blue), 0 to 100 % B (gradient 35 min), λ = 220 nm. Please note that a HIC run with water only was performed and the gained absorption values were subtracted from the absorption values of the analytes. Scheme was created with biorender.com.

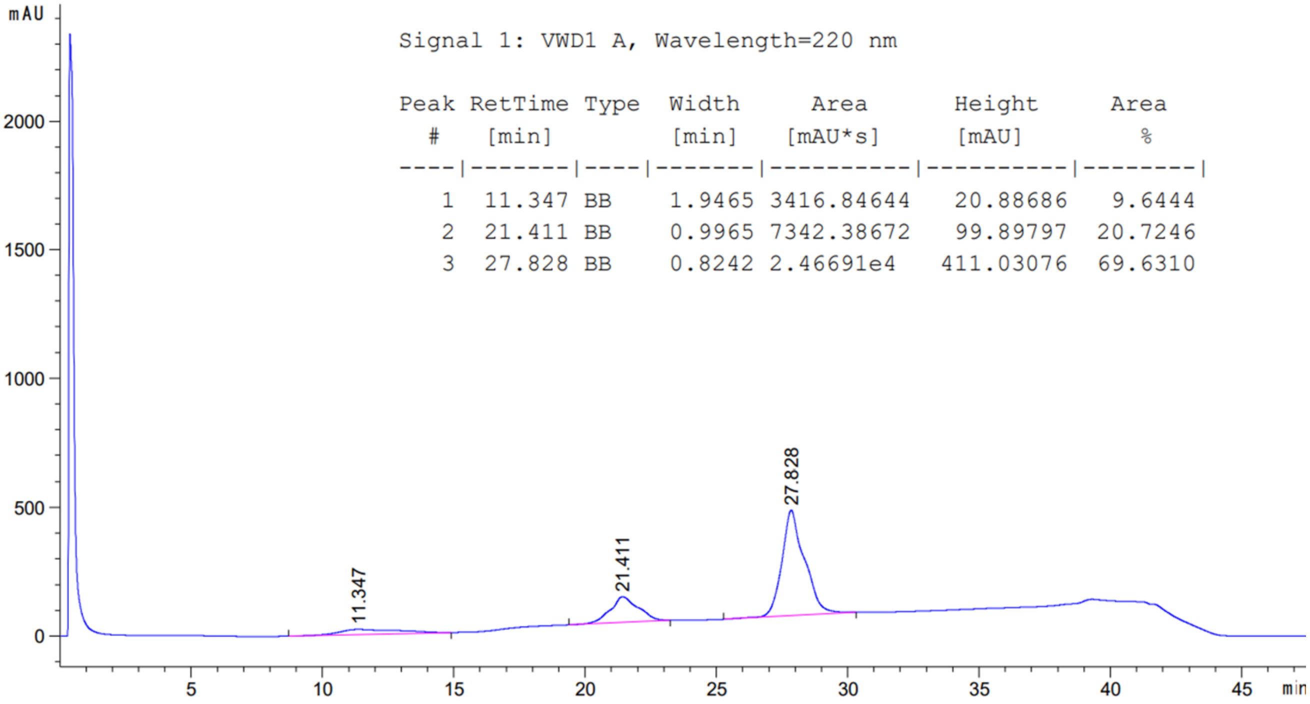

Supplementary Figure 28: HIC trace of Fc-MMAE **3** with integrated peaks, 0 to 100 % B (gradient 35 min), λ = 220 nm.

Drug-to-protein ratio (DPR) was determined from the relative ratios of areas under the curves (AUC). Peak #1 (t_R = 11.347 min) corresponds to non-functionalized Fc **1**, Peak #2 (t_R = 21.411 min) to Fc carrying one MMAE and Peak #3 (t_R = 27.828 min) to fully-functionalized Fc **3**. Determination of DPR was carried out using the following equation:

$$DPR = \sum_i AUC_i * n_i \;=\; (0.096 * 0) + (0.207 * 1) + (0.696 * 2) = \; 1.599$$

For further analysis reducing SDS-PAGE (Supplementary Figure 29) was performed. It displays a single band for Fc **1** corresponding to the reductive cleavage of disulfide bonds. Conjugation of MMAE **2** did not result in a significant shift.

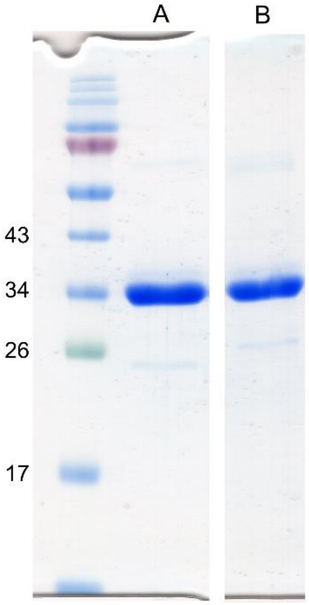

Supplementary Figure 29: Reducing SDS-PAGE of Fc **1** (lane A, M_w = 53.2 kDa) and Fc-MMAE **3** (lane B, M_w = 56.2 kDa (fully-functionalized), M_w = 54.7 kDa (mono-functionalized)).

2.5 Transglutaminase-Catalyzed Conjugation of Dextran

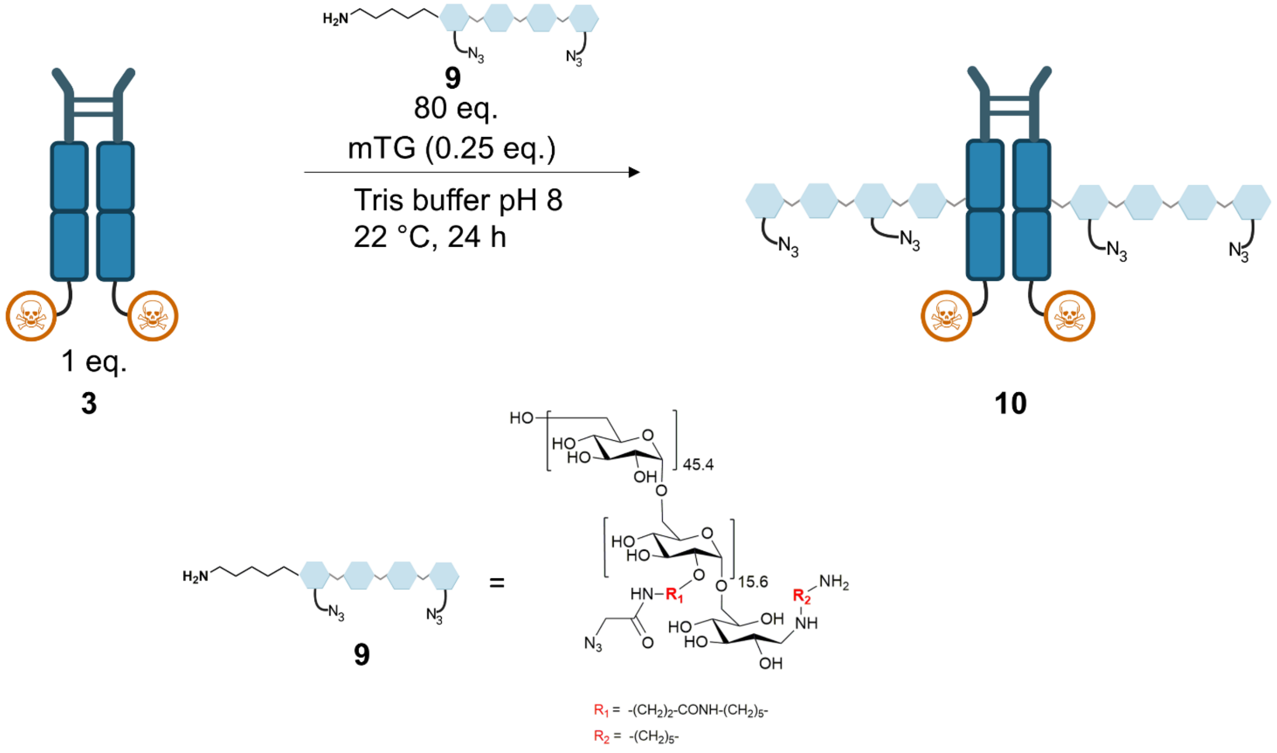

Supplementary Figure 30: Transglutaminase-mediated conjugation of dextran **9** to Fc-MMAE **3**. Scheme was created with biorender.com.

Reaction control was performed via reducing SDS-PAGE (Supplementary Figure 31). As could be also observed in a previous publication (Schneider et al., 2019b), mTG-mediated conjugation on Fc led to a product which shows no visible reduction in size under reducing conditions. This might be the result of mTG-mediated crosslinking between glutamine and lysine residues, respectively, during the long incubation time of 24 hours. Conjugation of dextran to Fc results in a smear over the protein in the gel, which is characteristic for dextran conjugation(Schneider et al., 2019a;Schneider et al., 2019b).

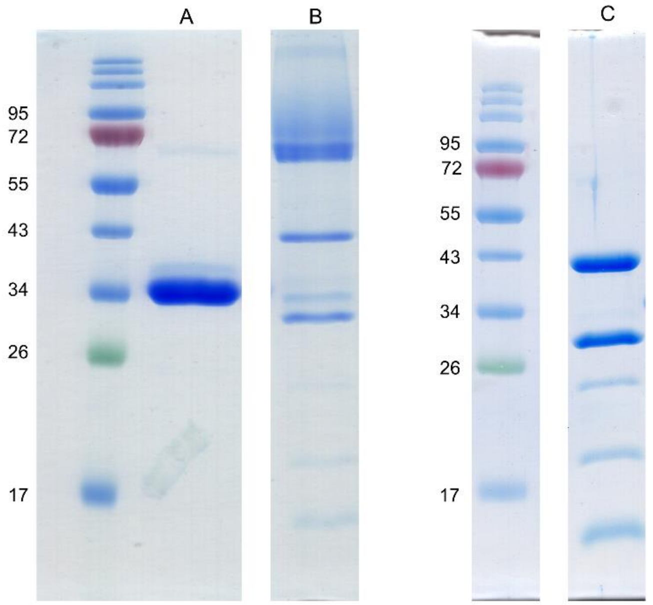

Supplementary Figure 31: Reducing SDS-PAGE of Fc-MMAE **3** (lane A, M_w = 56.2 kDa) and dextran-modified product **10** (lane B, M_w = 83.8 kDa). Please note that the probe was not purified by Protein A affinity chromatography, wherefore trypsin activated transglutaminase (lane C) was still present in lane B which results in additional bands.

Synthesis of Fc-dextran **22** was performed analogously to the synthesis of dextran-modified Fc-MMAE **10**:

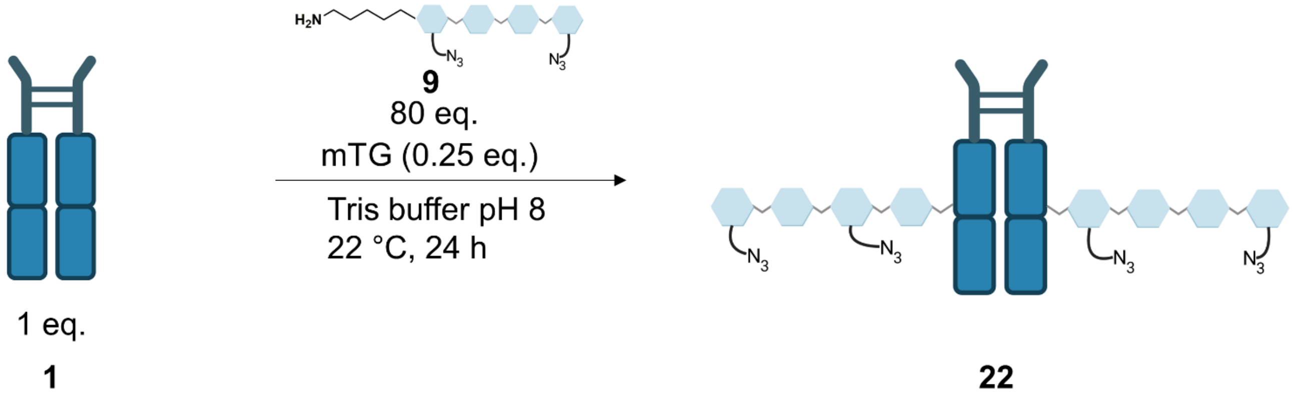

Supplementary Figure 32: Transglutaminase-mediated conjugation of dextran **9** to Fc-MMAE **1**. Scheme was created with biorender.com.

Reaction control was performed via reducing SDS-PAGE (Supplementary Figure 33). Like in the previous reaction (Supplementary Figure 31), the product Fc shows no size reduction under the reducing conditions and, furthermore, dextran conjugation resulted in the presence of smear above the protein band.

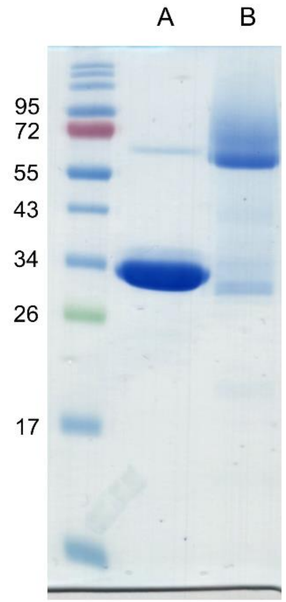

Supplementary Figure 33: Reducing SDS-PAGE of Fc **1** (lane A, M_w = 53.2 kDa) and dextran-modified product **22** (lane B, M_w = 80.8 kDa).

2.6 CuAAC-Catalyzed Cycloaddition

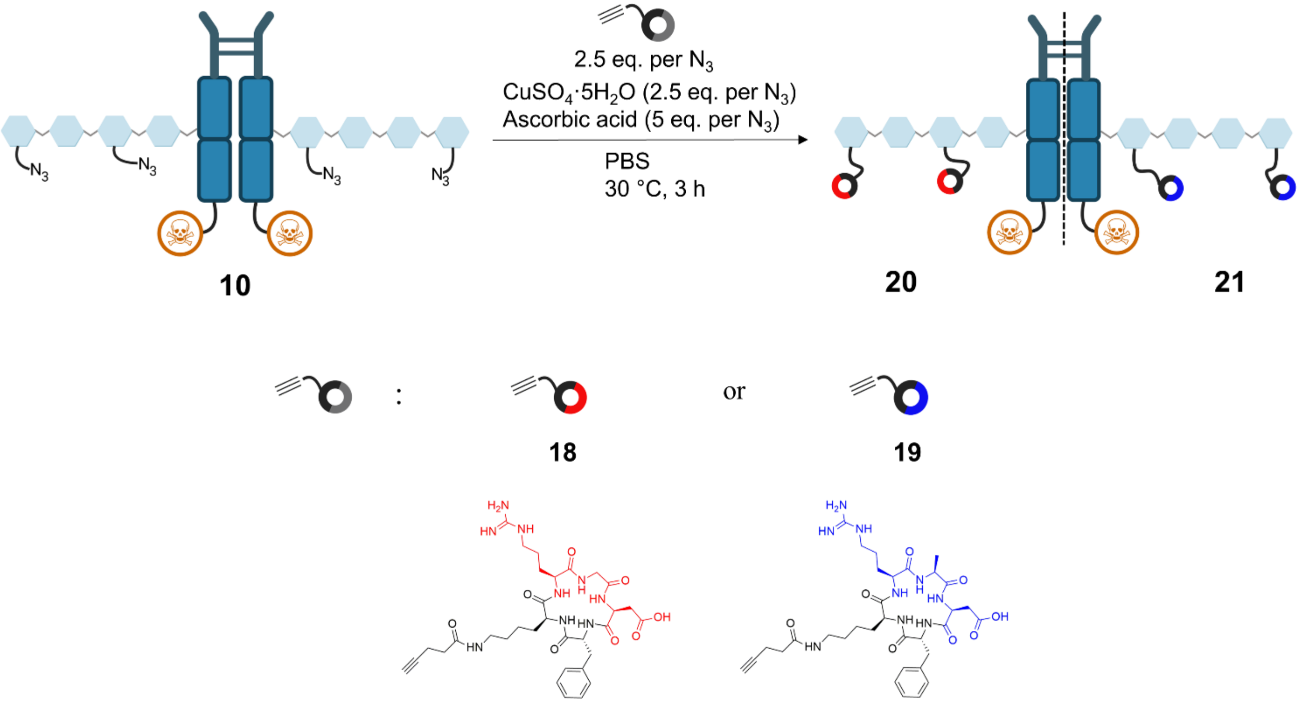

Supplementary Figure 34: CuAAC-catalyzed conjugation of alkyne-modified **18** and **19**, respectively, to Fc-dextran hybrid **10**. Scheme was created with biorender.com.

Reaction success was confirmed with SDS-PAGE (Supplementary Figure 35), which displays a shift of the dextran-protein smear to higher molecular weight compared to the educt **10** (Supplementary Figure 31).

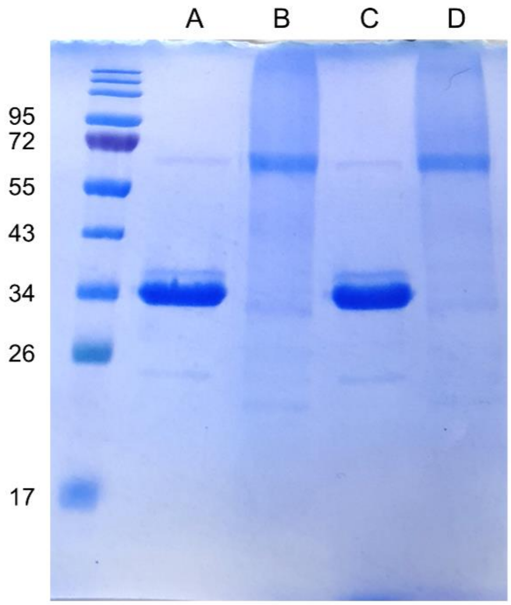

Supplementary Figure 35: Reducing SDS-PAGE of Fc **1** (lane A and C, M_w = 53.2 kDa), RGD-decorated **20** (lane B, M_w = 105.2 kDa) and RAD-decorated **21** (lane D, M_w = 105.7 kDa).

Synthesis of RGD-decorated **23** and RAD-decorated **24**, respectively, was performed in the same fashion:

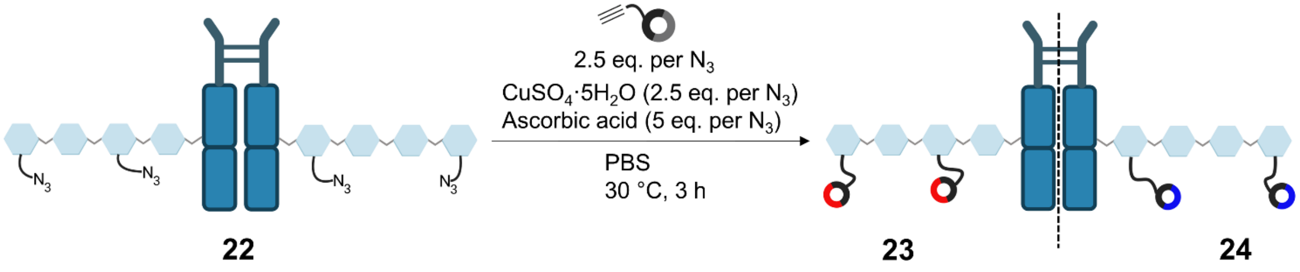

Supplementary Figure 36: CuAAC-catalyzed conjugation of alkyne-modified **18** and **19**, respectively, to Fc-dextran hybrid **22**. Scheme was created with biorender.com.

SDS-PAGE analysis (Supplementary Figure 37) also shows a shift in the smear upon CuAAC-mediated conjugation of RGD and RAD, respectively.

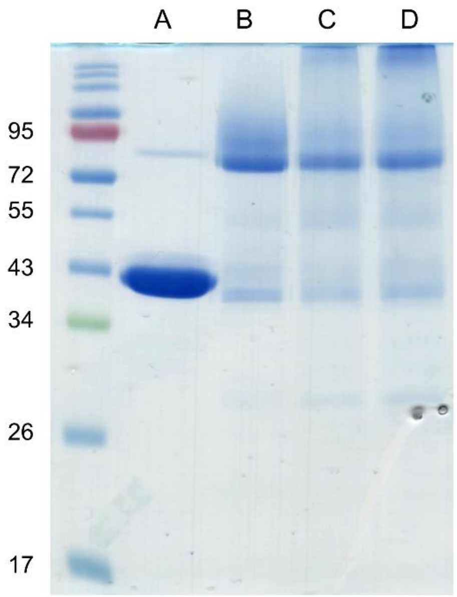

Supplementary Figure 37: Reducing SDS-PAGE of Fc **1** (lane A, M_w = 53.2 kDa), Fc-dextran **22** (lane B, M_w = 80.8 kDa), RGD-decorated **23** (lane C, M_w = 102.2 kDa) and RAD-decorated **24** (lane D, M_w = 102.7 kDa).

3 Thermal Shift Assay

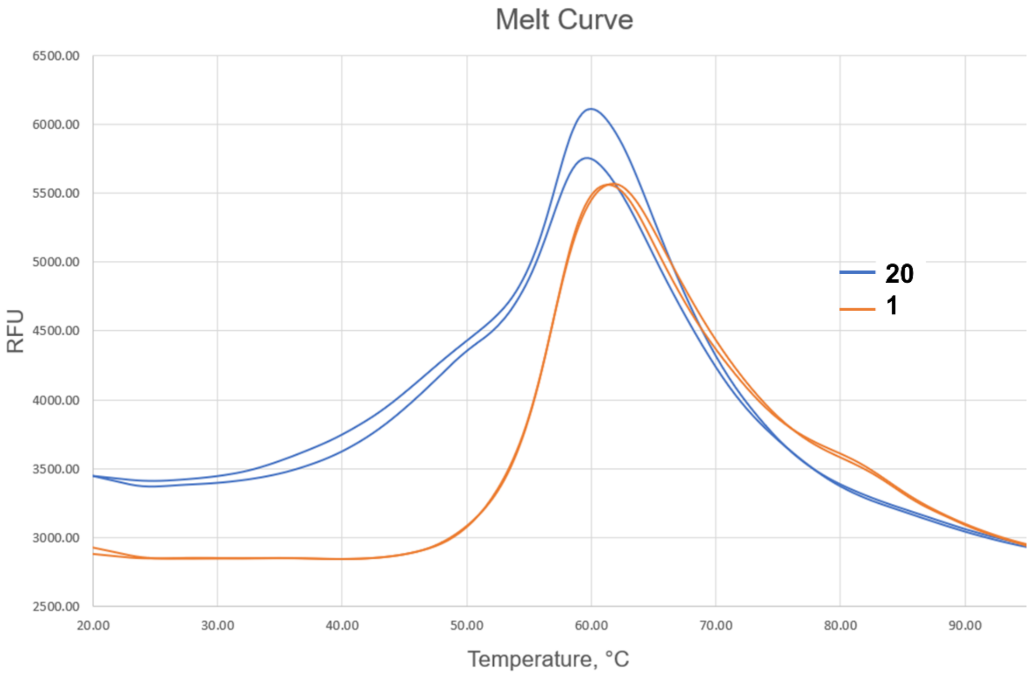

Supplementary Figure 38: Melt curve of RGD-decorated **20** and parent Fc **1**.

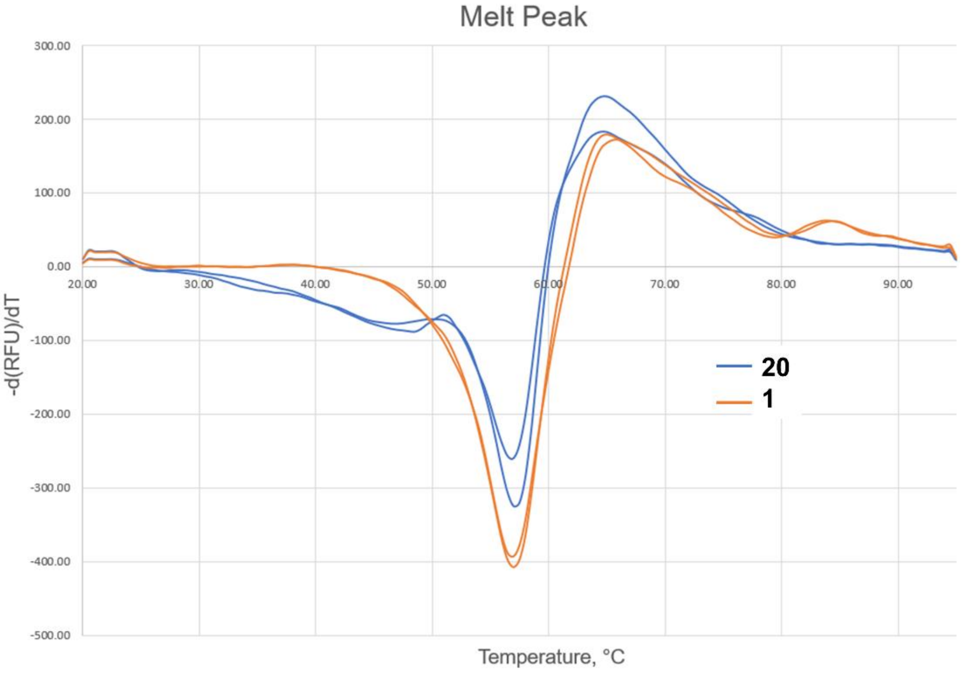

Supplementary Figure 39: Melt peak of RGD-decorated **20** and parent Fc **1**.

4 K$_D$ on U87MG cells

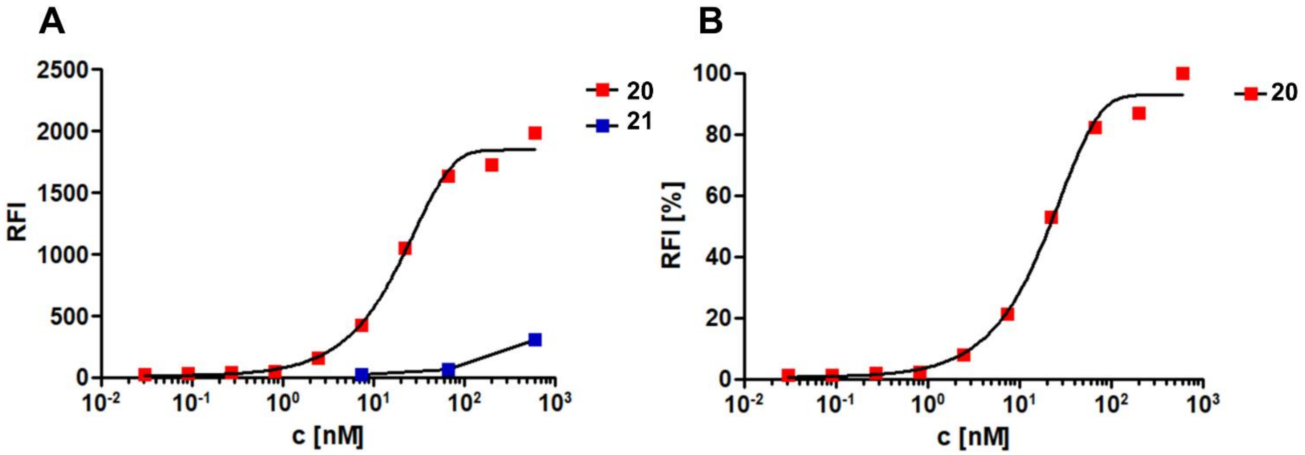

Supplementary Figure 40:A: Apparent K$_D$ determination of RGD-decorated **20** and RAD-decorated **21** on U87MG cells. **B**: K$_D$ determination of RGD-decorated **20**, where the relative fluorescence intensity (RFI) is displayed in percentages (100 % = highest value for 600 nM construct **20**).

5 Literature

Schneider, H., Deweid, L., Pirzer, T., Yanakieva, D., Englert, S., Becker, B., et al. (2019a). Dextramabs: A Novel Format of Antibody-Drug Conjugates Featuring a Multivalent Polysaccharide Scaffold. 8**,** 354-357. doi:https://doi.org/10.1002/open.201900066

Schneider, H., Yanakieva, D., Macarrón, A., Deweid, L., Becker, B., Englert, S., et al. (2019b). TRAIL-Inspired Multivalent Dextran Conjugates Efficiently Induce Apoptosis upon DR5 Receptor Clustering. *ChemBioChem* 20**,** 3006-3012. doi:https://doi.org/10.1002/cbic.201900251
